# High-Resolution Mutation Mapping Reveals Parallel Experimental Evolution in Yeast

**DOI:** 10.1371/journal.pbio.0040256

**Published:** 2006-07-25

**Authors:** Ayellet V Segrè, Andrew W Murray, Jun-Yi Leu

**Affiliations:** **1**Department of Molecular and Cellular Biology, Harvard University, Cambridge, Massachusetts, United States of America; University of DublinIreland

## Abstract

Understanding the genetic basis of evolutionary adaptation is limited by our ability to efficiently identify the genomic locations of adaptive mutations. Here we describe a method that can quickly and precisely map the genetic basis of naturally and experimentally evolved complex traits using linkage analysis. A yeast strain that expresses the evolved trait is crossed to a distinct strain background and DNA from a large pool of progeny that express the trait of interest is hybridized to oligonucleotide microarrays that detect thousands of polymorphisms between the two strains. Adaptive mutations are detected by linkage to the polymorphisms from the evolved parent. We successfully tested our method by mapping five known genes to a precision of 0.2–24 kb (0.1–10 cM), and developed computer simulations to test the effect of different factors on mapping precision. We then applied this method to four yeast strains that had independently adapted to a fluctuating glucose–galactose environment. All four strains had acquired one or more missense mutations in *GAL80,* the repressor of the galactose utilization pathway. When transferred into the ancestral strain, the *gal80* mutations conferred the fitness advantage that the evolved strains show in the transition from glucose to galactose. Our results show an example of parallel adaptation caused by mutations in the same gene.

## Abstract 2

An array hybridization method enables genetic mapping via linkage analysis; applied here this new method shows parallel adaptation to a fluctuating glucose-galactose environment evidenced by mutations in the *GAL80* gene.

## Introduction

Characterizing the genetic changes that underlie evolutionary adaptation is important for understanding the emergence of new phenotypes. Experimental evolution makes it possible to follow the evolutionary history of populations exposed to known selective pressures. Moreover, the reproducibility of evolutionary paths can be explored by comparing identical, independent experiments. Such studies are beginning to shed light on the genetic basis of evolutionary adaptation [ 1– 4], but many questions remain open, such as how rare gain-of-function mutations are relative to loss-of-function ones, and how often similar phenotypic adaptations are the result of similar genetic changes. A major challenge is finding the adaptive (beneficial) mutations without having to make prior assumptions about their type or site.

Several strategies have been used to search for mutations associated with evolved traits. These include sequencing candidate genes [ 5– 7], tracking the insertion sites of mobile genetic elements [ 8– 10], partial- or whole-genome sequencing [ 1, 11– 13], gene expression profiling [ 2, 14], identifying large chromosomal rearrangements [ 8, 15], and linkage analysis [ 16– 18]. Some of these approaches rely on the assumption that mutations found repeatedly in several independently evolved populations are likely to be beneficial. Ultimately, the effects of the mutations on the evolved phenotypes have to be verified experimentally [ 3, 4, 19].

Linkage analysis is the least biased and most general method for finding adaptive mutations in a background of neutral ones. It relies on linkage between the mutations that produce the phenotype of interest and neutral genetic markers (DNA polymorphisms) that can be easily followed, and thus makes no assumptions about the nature or locations of the adaptive mutations [ 20, 21]. Such analyses are often applied to progeny (segregants) from a cross between two strains that differ for both the selected trait and the genetic markers. Advances in genome technology have enabled simultaneous genotyping of thousands of DNA polymorphism markers by hybridizing genomic DNA to oligonucleotide arrays [ 22, 23]. This has led to better genome coverage and mapping resolution, as demonstrated on several traits in budding yeast, including growth at high temperature and sporulation efficiency [ 22, 24, 25]. However, such quantitative trait mapping methods are laborious and expensive for mapping multiple traits or multiple strains (e.g., strains evolved in parallel experiments), as they usually require the genotyping of multiple individual segregants for each strain or trait being mapped. One solution is to mix DNA from many individuals expressing the trait of interest, and genotype it as a pool (selective DNA pooling; [ 26]). A variety of pooled DNA genotyping methods have been used in association studies in humans [ 27– 30], as well as in quantitative trait locus (QTL) mapping in plants and animals, where experimental crosses are possible [ 31– 36].

Here we map mutations in the budding yeast, *Saccharomyces cerevisiae,* which we use as a model organism to study the genetic basis of experimentally evolved traits ([ 37]; see also [ 4]). To overcome the limitations described above, we used high-density oligonucleotide arrays to genotype a single large pool of segregants that express the trait of interest, an approach also used in plants [ 33]. This strategy reduces the number of microarrays needed for mapping, and increases mapping resolution due to the wide variety of recombination breakpoints present in a large pool of segregants. We tested and optimized our method on five known genetic loci and developed computer simulations to test the effect of various factors on mapping precision. We then applied it to four yeast strains that have been evolved in an environment where they were exposed to a regular alternation of carbon sources. The adaptive phenotype was mapped to the same locus in all four strains. We identified the adaptive mutations in the mapped regions and experimentally verified their contribution to the evolved trait.

## Results

### A Bulk Segregant Mapping Method

**Figure 1:**
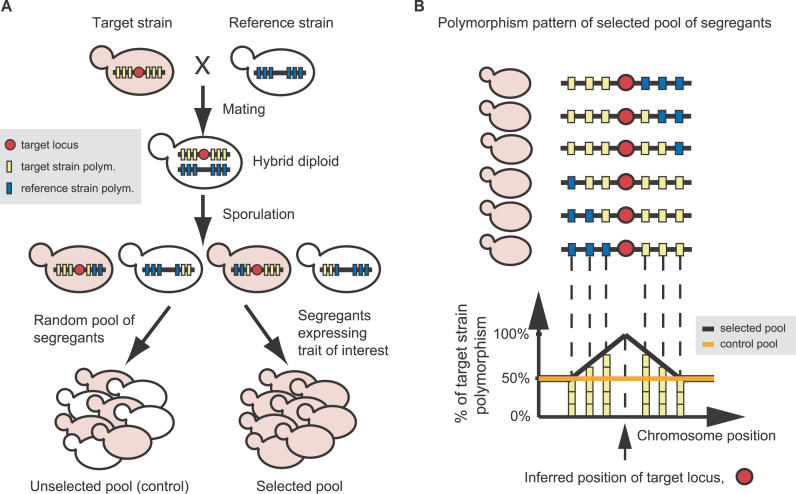
Schematic Description of the Pooled Mapping Method Applied to a Single Pool of Segregants in Budding Yeast (A) A strain expressing the trait of interest (target strain) is crossed to a highly polymorphic strain that lacks the trait (reference strain). The DNA polymorphisms between the strains, represented by yellow squares for the target strain's genotype and blue squares for the reference's genotype, are the genetic markers used for the linkage analysis. After mating, the hybrid diploid is sporulated, yielding a pool of haploids (segregants) that are genetically diverse due to random recombination along the chromosomes. A large pool of segregants that express the trait of interest (cells with red background) is selected, enriching for segregants that carry the alleles that give rise to the trait of interest (target locus; represented by red circle). As a control, a pool of segregants of comparable size is randomly collected. For simplicity, only a single chromosome is shown. (B) The location of the target locus is inferred by a genome-wide comparison of the fraction of the target strain's genotype (yellow squares) within the selected pool to that within the control pool. The genomic DNA of the selected and control pools are extracted and their patterns of polymorphisms along the genome are analyzed. At polymorphic sites that are unlinked to the target locus, half of the segregants in the selected pool are expected to display the target strain's genotype and half the reference strain's genotype. However, at linked polymorphic sites, the fraction of selected segregants that carry the target strain's genotype (black line) should be higher than 50% and inversely proportional to the distance between the polymorphic site (squares) and the target locus (red circle). For the control pool, the genotype of both parental strains should be equally represented throughout the entire genome (orange line). The target loci lie in chromosome regions where the target strain's genotype is significantly overrepresented in the selected pool relative to the control pool.

A schematic description of the mapping method is presented in Figure 1. Briefly, a haploid yeast strain that expresses the trait of interest (the target strain) is crossed to a reference strain that lacks the trait and differs from the target strain at thousands of polymorphic sites ( Figure 1A). The hybrid diploid is then sporulated (undergoes meiosis), giving rise to a diverse pool of recombinant haploid progeny (segregants) that contain different combinations of their parents' genomic DNA. A large pool of segregants that express the trait of interest is selected from the progeny (the selected pool); as a control, a random pool of similar size is collected without selecting for the trait (the control pool). DNA from each pool is hybridized to an oligonucleotide microarray that detects the polymorphic sites. If it is difficult to select simultaneously for multiple segregants that express the trait, single progeny can be screened individually for the phenotype and later pooled for the linkage analysis. For each locus, the extent of hybridization reveals the fraction of the DNA that is derived from the target strain ( Figure 1B); regions where the target strain's genotype is overrepresented in the selected pool relative to the control pool are predicted to contain mutations that contribute to the trait of interest (target loci).

### Genetic Map Construction for Linkage Analysis

The first step in mapping is finding loci that are polymorphic between the target and reference strains. We identified these by hybridizing the genomic DNA of the target and reference strains separately onto high-density oligonucleotide arrays. Oligonucleotides (features) that hybridized significantly more strongly to DNA of the target strain than to that of the reference strain were the polymorphic features considered in this study (single-feature polymorphisms [SFPs]). We identified SFPs with a detection algorithm that uses a one-tailed two-sample *t* test (see Materials and Methods) and estimated its sensitivity and specificity using two strains (S288c and YJM789) whose genomic DNA sequences are known (see Materials and Methods). We identified 4,438 S288c/YJM789 SFPs out of 12,602 true SFPs at a *p* value of 10 ^−6^, yielding a true-positive rate of 35.22% (fraction of the true polymorphic features that are scored as polymorphic) at an estimated false discovery rate (FDR) of 6.74% (fraction of detected SFPs that are not truly polymorphic) ( Figure S1). At this *p* value, we detect 45% of the SFPs where the sequence difference between the two strains lies in the central 15 bases of the 25-base oligonucleotide on the array. We chose a *p* value cutoff of 10 ^−6^ for SFP identification as it gave the highest true-positive–to–false discovery rate ratio of the cutoffs tested.

### Mapping Method Tested on Five Known Genes

We asked whether we could use pools of segregants from single crosses to map known genetic loci. We chose W303 and SK1 as the target and reference yeast strains as they are widely used in laboratory studies and a high level of polymorphism was reported between them [ 23]. Using our SFP detection algorithm, we identified 10,330 W303/SK1 SFPs at an estimated FDR of 2.9%, resulting in an average marker density of 1 SFP per ˜1.1 kb or ˜0.4 cM (the distribution of distances between SFPs is shown in Figure S2). These SFPs made up the genetic map used for the linkage analyses in this work.

**Figure 2:**
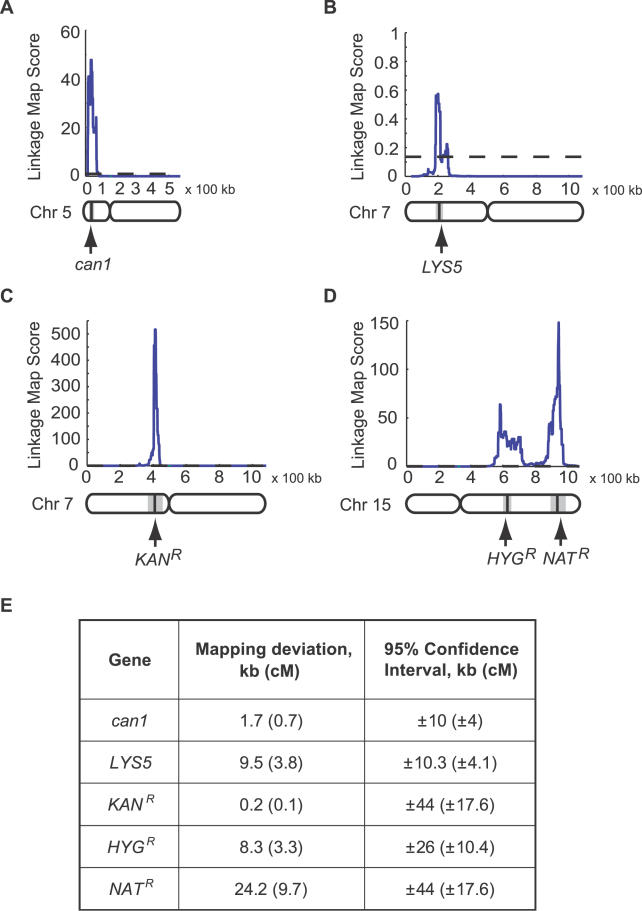
Successful Mapping of Known Genes Two separate pools of ˜10 ^7^ W303/SK1 segregants, one resistant to canavanine that grew without lysine *(can1, LYS5)* and one resistant to geneticin, hygromycin, and nourseothricin *(KAN ^R^, NAT ^R^, HYG ^R^),* were selected for and mapped. The five genes mapped are: (A) *can1,* centered on Chromosome 5, position 32.6 kb; (B) *LYS5,* on Chromosome 7, position 215.7 kb; (C) *KAN ^R^,* on Chromosome 7, position 413.4 kb; (D) *HYG ^R^,* on Chromosome 15, position 619.1 kb; and *NAT ^R^,* on Chromosome 15, position 960.6 kb. The LMS was calculated across the whole genome for each pool (shown in Figure S3) and is plotted here along the chromosomes that carry the five target genes, as a function of chromosome position in 100-kb units. The five peaks that correspond to the five target genes all fell above the significant peak thresholds estimated at 99% confidence for each selected pool (horizontal dashed lines, which are so close to the *x*-axis as to be invisible in [C] and [D]). The arrows mark the actual center of the target genes, the solid lines within the drawn chromosomes mark the predicted center of the genes, and the grey boxes within the chromosomes mark the 95% confidence intervals estimated with simulations (see Materials and Methods). The peak for *LYS5* is low relative to the significant peak cutoff, because of the low local SFP density (see Figure S3G– S3I for discussion). Note that the scale of the *y*-axis is different in the four panels ( Protocol S1). (E) The mapping deviations of the genes' predicted centers from their actual centers and their 95% confidence intervals. Their corresponding average genetic distance in cM is written in parentheses. All five genes were found within their 95% confidence intervals.

We tested the performance of our method by mapping one metabolic and four drug resistance genes whose chromosomal locations are known (details in Materials and Methods). The target strain was a derivative of W303 that carries alleles that confer resistance to four drugs, canavanine *(can1),* geneticin *(KAN ^R^),* hygromycin *(HYG ^R^),* and nourseothricin *(NAT ^R^),* and that can produce lysine *(LYS5).* It was crossed to the reference strain, a derivative of SK1 that is sensitive to all four drugs and cannot make lysine *(lys5),* and the hybrid diploid was sporulated. To simultaneously map *LYS5* and *can1,* we selected approximately 10 ^7^ segregants that grew in liquid medium lacking lysine and containing canavanine. To map *KAN ^R^, HYG ^R^,* and *NAT ^R^,* we selected a second pool of approximately 10 ^7^ segregants in liquid medium containing geneticin, hygromycin, and nourseothricin (Clonat). A control pool of segregants of comparable size was isolated in rich medium without drugs. The genomic DNA of each selected and control pool was hybridized to four identical arrays. The hybridization intensities of the selected and control pools and the target strain at the W303/SK1 SFPs were converted into a linkage map score (LMS) by analyzing the intensities across a moving window along the genome that included 50 SFPs (see Materials and Methods). This score reflects the probability that a given chromosomal region is linked to the selected trait (for further discussion, see Protocol S1). We used simulations to estimate the minimum LMS that we considered significant. The order of the SFPs was scrambled 1,000 times, for each ordering we recorded the highest LMS found across the genome, and we considered peaks in the unpermuted data significant only if they exceeded the 99th percentile of the ranked maximum scores from the permuted simulations (see Materials and Methods). Figure 2 shows the LMS for the chromosomes containing the mapped loci, and Figure S3 shows the LMS across the entire genome.

A total of six peaks were found above the cutoff: two for the *can1, LYS5* pool and four for the *KAN ^R^, HYG ^R^,* and *NAT ^R^* pool. Of the six peaks, five corresponded to the five selected genes ( Figure 2). We used mapping deviation, the distance between the center of a mapped peak and the center of the linked target gene, as a measure of mapping precision. In addition, we estimated 95% confidence intervals for each mapped locus using computer simulations (see Materials and Methods). The mapping deviations of the five genes, 0.2, 1.7, 8.3, 9.5, and 24 kb (˜0.1–10 cM) for *KAN ^R^, can1, HYG ^R^, LYS5,* and *NAT ^R^,* respectively, all fell within their 95% confidence intervals, which ranged from ±10 to ±44 kb (±4 to ±18 cM). The mapping deviations are robust to the array preprocessing method used (see Tables S1 and S2, and Figure S4). This test case demonstrates that genes proximal to the centromere or telomere *(can1* and *KAN ^R^)* can be mapped at high resolution, and that two genes on the same chromosome arm can be easily separated from each other *(HYG ^R^* and *NAT ^R^).* The LMS is influenced by several factors, including SFP density, local recombination frequency, and the variance of the hybridization to individual probes on the array. As a result, the differences between the LMSs of the different peaks do not reflect the quantitative contribution of the different loci to the selected phenotype (see Protocol S1).

Aside from one potential false-positive peak found on Chromosome 10 at position 146.3 ± 30 kb, the signal-to-noise levels of the LMS was high ( Figures S3 and S5). One possible interpretation for the extra peak is that it has uncovered real DNA differences between the target (W303) and reference (SK1) strains that contribute to the resistance to one or more of the drugs used for the selection. The failure to observe this peak in the *can1, LYS5* pool supports this hypothesis, although we found no obvious candidates for genes whose polymorphisms might contribute to drug resistance in the 95% confidence interval on Chromosome 10.

To assess the consistency of our mapping precision, *KAN ^R^, HYG ^R^,* and *NAT ^R^* were mapped in two additional selected pools derived from different initial W303/SK1 hybrid diploids, yielding a total of three completely independent mapping experiments (unpublished data). The genes' mean mapping deviations and their standard deviations are 7.5 ± 6.4 kb, 4.1 ± 3.6 kb, and 14.9 ± 8.6 kb, respectively, with a total mean mapping deviation of 8.8 kb.

For some organisms or phenotypes, selecting for 10 ^7^ segregants that express the trait of interest is impractical. We therefore asked whether we could map the *KAN ^R^, HYG ^R^,* and *NAT ^R^* alleles with 10 ^4^ or 100 segregants. Even with 100 segregants, the three drug-resistance genes were mapped to within 50 kb of their correct locations ( Table 1; see Figure S3 for whole-genome maps). No significant correlation was seen between the mapping precision of the three genes and pool size (100, 10 ^4^, and 10 ^7^ segregants) ( *p* > 0.6, *t* test).

**Table 1:**
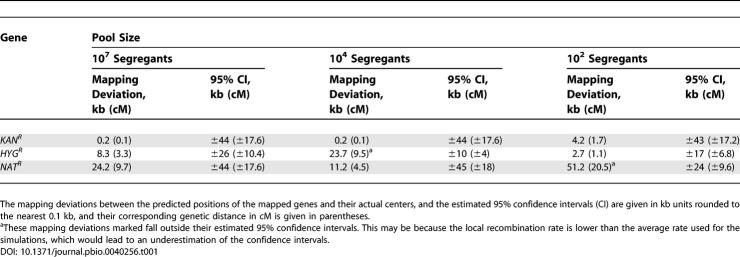
Mapping Precision as a Function of Segregant Pool Size

A variety of factors could cause alleles that contribute to a trait to be absent from some members of a pool that had been selected to express the trait strongly. To examine the robustness of our method to such deviations, we tested whether three drug-resistance genes could be mapped when their enrichment level in the selected pool is close to 70%–75% instead of 90%–100% as with our test case. Thus, we mixed equal amounts of DNA from a pool of segregants selected for resistance to geneticin, hygromycin, and nourseothricin and a control, unselected pool, yielding an approximately 3:1 ratio of target strain polymorphisms to reference polymorphisms in regions linked to the drug resistance genes. Although the LMSs were greatly reduced, we still observed three peaks that corresponded to the three selected genes, demonstrating that we can map alleles that are absent from a substantial fraction of the selected pool ( Figure S3J). In addition, the signal-to-noise ratio of the LMSs was still substantially high ( Figure S5).

### Computer Simulations of the Mapping Process

**Figure 3:**
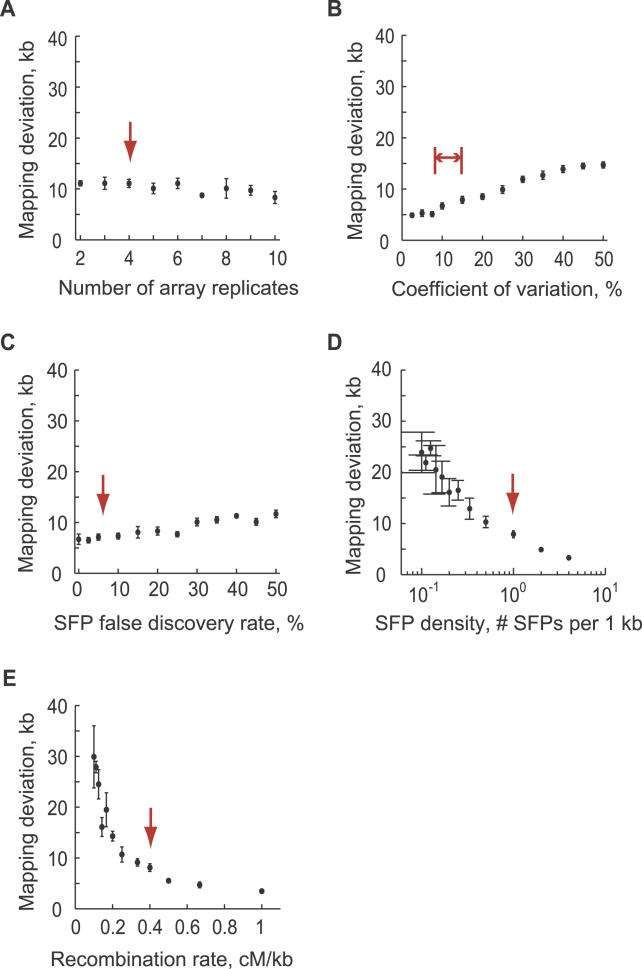
Computer Simulations Evaluate the Effect of Different Factors on Mapping Precision The absolute distance of the predicted position of a simulated target locus from its real position (mapping deviation) is plotted as a function of (A) number of array replicates, (B) coefficient of variation (standard deviation divided by the mean) of hybridization intensities, (C) SFP FDR, (D) SFP density, and (E) recombination rate. Aside from the varying factor, the parameters were set to the values observed in our test case ( Figure 2) and are marked on each panel with a red arrow. With the exception of (A), four replicate arrays were used for the selected and control pools, and eight replicates for the target strain. The mean coefficient of variation values of all our test case hybridizations at SFPs varied between 8%–15%. A smoothing window of 50 SFPs was used for all simulations except for SFP density, where a window of constant chromosome size (50 kb) was used. The mean and standard errors were computed from 5 repetitions of 1,000 simulation runs each for every datapoint. Error bars that are not visible are smaller than the dot. Note the logarithmic scale of the *x*-axis of SFP density.

We developed a computer model that simulates the whole mapping process (see Materials and Methods) to assess the effect of experimental design (e.g., number of arrays), intrinsic genetic factors (e.g., recombination rate), and adjustable statistical parameters (e.g., *p* value cutoff for SFP detection) on mapping precision. Figure 3 presents the effect of the number of array replicates, noise levels between replicate intensities (coefficient of variation, the standard deviation divided by the mean), SFP FDR, SFP density (number of SFPs per 1 kb), and recombination rate on mapping deviation. Aside from the factor being varied, the parameters for the simulations were taken from our mapping experiments. The mapping deviations obtained with simulations are consistent with the experimentally observed mapping deviations for the five test case genes. Of the factors tested, SFP density and recombination rate displayed the strongest effect on mapping precision, with higher SFP density and higher recombination rate improving mapping precision. Even in regions with a ten-fold lower SFP density than average (˜99% of SFPs lie in denser regions; Figure S2) or a four-fold lower recombination rate than average, our simulations suggest that a gene can successfully be mapped, albeit with lower resolution. We tested the effect of SFP density in our experiments by excluding varying fractions of the SFPs that lay near *KAN ^R^* from our analysis, to create an SFP density that ranged from 1.8 to 0.2 SFPs/kb. The negative trend of mapping deviation as a function of SFP density seen in simulations (Pearson's correlation coefficient *R ^2^* = −0.96 for mapping deviation versus logarithm of SFP density) corresponded closely to that observed in this manipulation of our experimental data ( *R ^2^* = −0.95). As increased SFP FDR has little impact on mapping precision, especially in our observed experimental range (3%–7%), choosing a more liberal cutoff for identifying SFPs might help to map genes located in regions with low SFP density. In contrast, the number of array replicates and coefficient of variation between replicate intensities show only a weak effect on mapping precision, in particular in the ranges relevant for our study (two to four arrays, and coefficient of variation of 8%–15%). The simulations suggest that duplicate arrays for the selected and control pools suffice, which is in agreement with our mapping results of the test genes (see Table S3).

### Mapping an Experimentally Evolved Trait in Yeast

**Figure 4:**
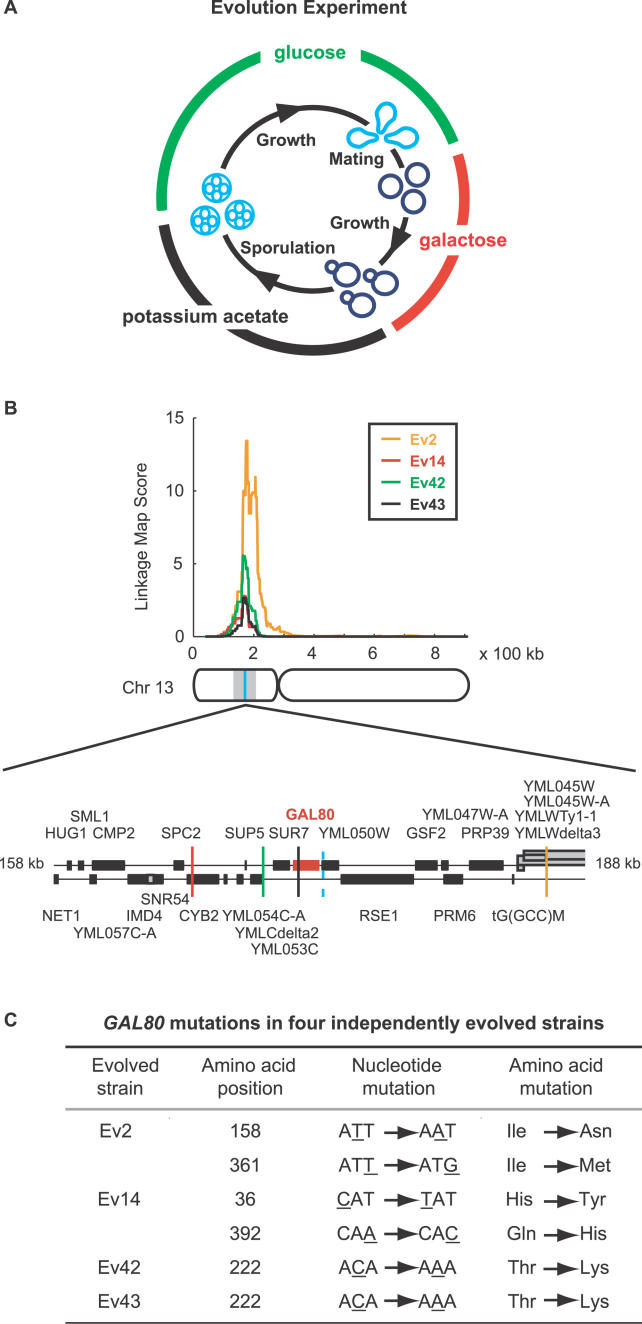
Four Evolved Strains That Independently Adapted to Glucose–Galactose Transition Acquired One or Two Missense Mutations in *GAL80* (A) A schematic description of the evolution experiment. In each cycle of evolution, haploid cells (light blue) were grown in glucose-containing media for 4 d, mated on YPD plates, and then transferred to galactose-containing media for 2 d. The diploid cells (dark blue) were then put through a sporulation cycle (meiosis) with potassium acetate as their carbon source. Each evolved population was put through 36 such cycles. (B) All four evolved strains were mapped to a single locus on Chromosome 13. The LMS is plotted as a function of chromosome position in 100-kb units for Chromosome 13, where a significant peak was detected in all four strains. The predicted peak centers and estimated 95% confidence intervals are: Ev2, 185.8 ± 27 kb (orange); Ev14, 165.8 ± 39 kb (red); Ev42, 169.8 ± 40 kb (green); and Ev43, 171.8 ± 36 kb (black). The solid cyan line within the schematic chromosome marks the mean of the four predicted peak centers (173.3 kb), and the grey box within the chromosome marks the mean estimated 95% confidence interval (±35.5 kb [±14.2 cM]). The genes that fall within a 30-kb interval around the mean peak center (cyan dashed line) are depicted below (black or gray boxes) with the *GAL80* gene colored in red (centered at 172.2 kb; gene coordinates taken from the *Saccharomyces* Genome Database). The peak centers of the four evolved strains are marked with lines color-coded according to their LMS plot. Figure S6 shows the LMS across the entire genome for this experiment. (C) *GAL80* was sequenced in the four evolved strains (Ev2, Ev14, Ev42, and Ev43) and in the ancestor, and the mutations found are presented at the nucleotide and amino acid levels. The mutated nucleotides are underlined. In addition, a deletion of a single T was found 96 nucleotides upstream to the translation start site of *GAL80* in Ev42. This mutation is unlikely to have a significant effect on the activity of Gal80, as the mutation in amino acid 222 alone was sufficient to recapitulate the adaptive phenotype (see Figure 5D). The *GAL80* sequence of the reference strain, SK1, is identical to that of the ancestor, W303, at the nucleotide level.

We used our mapping method to uncover the genetic basis of an experimentally evolved trait in yeast. We chose four W303 populations, derived from the same ancestor, that had been alternately grown in glucose- and galactose-containing media for 36 sexual cycles as part of a selection for altered mating preference [ 37] ( Figure 4A). A single haploid clone was chosen from each population after the 36th cycle of selection. After ˜700 generations all four strains had evolved to resume proliferation more rapidly than their ancestors when transferred from glucose- to galactose-containing medium (see below). *GAL3,* which encodes a coinducer of the galactose pathway [ 38], was found to be overexpressed three- to five-fold in the evolved strains compared with the ancestor when grown in medium with glucose as the sole carbon source (unpublished data).

**Figure 5:**
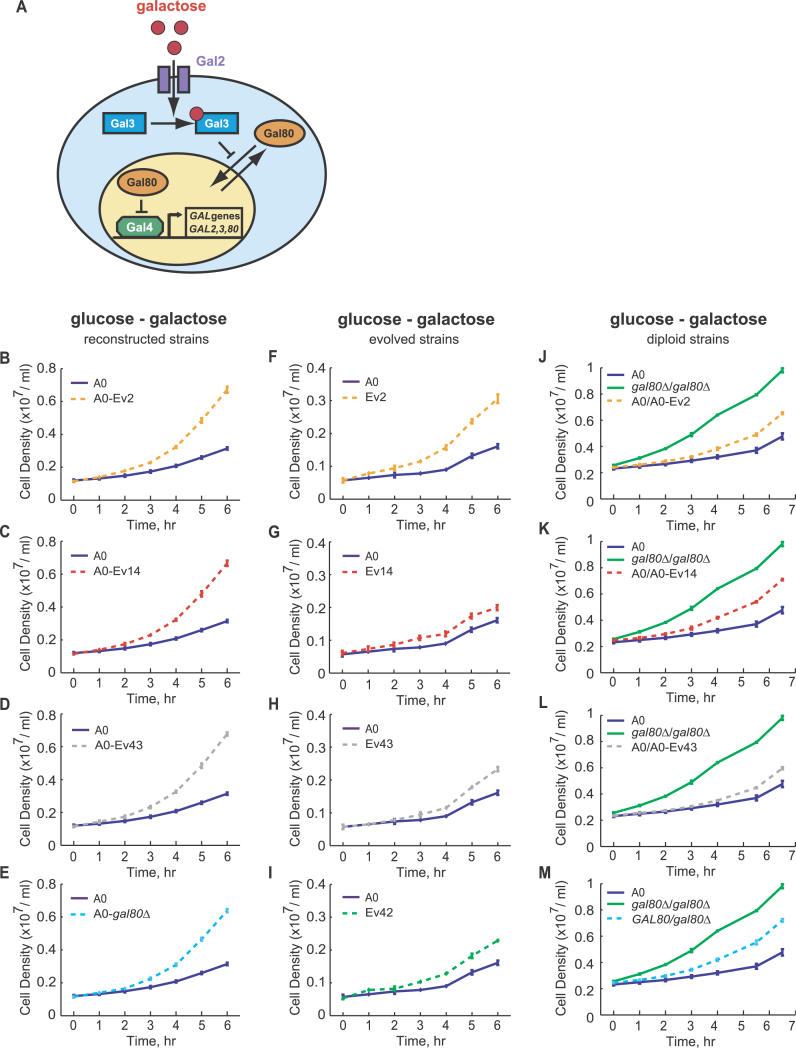
Transformation of *gal80* Mutations into the Ancestral *GAL80* Reconstructs the Adaptive Phenotype (A) A schematic depiction of the regulation of the galactose utilization pathway. In the absence of galactose, Gal80 inhibits the transcriptional activator, Gal4, by binding to Gal4 in the nucleus. When galactose is present it enters the cell through Gal2 transporters and binds Gal3, a coinducer of the pathway, which in turn binds Gal80 in the cytoplasm, sequestering Gal80 away from the nucleus. This relieves the repression of Gal4 allowing it to induce the transcription of genes required for galactose uptake and catabolism ( *GAL* genes), including *GAL2, GAL3, GAL80,* and the genes encoding the enzymes of galactose catabolism [ 38]. A similar phenotype is obtained through loss-of-function of the repressor, *GAL80.* (B–E) The *gal80* mutations confer a fitness advantage in transfers of exponentially growing haploid cells from glucose- to galactose-containing medium, but not in transfers in which the carbon source does not change ( Figure S7). Three different sets of *gal80* mutations in the coding region were transformed into the ancestral *GAL80* gene in an ancestral haploid strain (A0) (Ev2 indicates the mutation is from evolved culture 2, etc.). Ev42 and Ev43 have the same mutation in the coding region. Cell density (OD) was measured for each of these strains and for the ancestor and a *GAL80* knockout strain following transfer from glucose- to galactose-containing medium. (F–I) The four evolved strains are more fit than their ancestor (A0) when transferred from glucose- to galactose-containing medium. Cell density (OD) was measured for the haploid evolved strains Ev2, Ev14, Ev43, and Ev42, and their ancestor, following transfer from medium containing only glucose to medium containing galactose. (J–M) The *gal80* mutations have a semidominant effect when present in one copy in the ancestral diploid strain following transfer from glucose- to galactose-containing medium. The cell density (OD) of ancestral diploids carrying one copy of the *gal80* mutations from either Ev2, Ev14, or Ev43 were compared to that of an ancestral diploid (A0) and a diploid lacking both copies of *GAL80 (gal80Δ/gal80Δ),* following transfer from glucose- to galactose-containing medium. As a control, an ancestral diploid was made hemizygous for *GAL80 (M; GAL80/gal80Δ).* Mean cell density and a standard deviation from at least three independent cultures were plotted for each datapoint for (B–M). Error bars that are not visible are smaller than the datapoint.

We used *GAL3* as a reporter gene to select for segregants from crosses between the evolved strains and SK1 (Ev/SK1) that express the adaptive phenotype. For each of the four evolved strains, about 10 ^4^ Ev/SK1 segregants that expressed high levels of Gal3 fused to a green fluorescent protein (GFP) were selected using flow cytometry (see Materials and Methods). The genomic DNA of the control and selected pools were hybridized onto two or three replicate arrays each and the LMS was calculated for the entire genome ( Figure S6). In all four strains, the adaptive phenotype was mapped to the same region on Chromosome 13 with a mean peak center of 173.3 kb and a mean 95% confidence interval of ±36 kb (±14 cM; Figure 4B). An additional peak linked to *GAL3-GFP* on chromosome 4 was found for strain Ev2 because the Ev2/SK1 hybrid diploid was heterozygous for the *GAL3*- *GFP,* with the one copy of *GAL3*- *GFP* lying on the chromosome derived from the evolved strain, while the other strains were homozygous for *GAL3*- *GFP* ( Figure S6).

*GAL80,* which encodes the key repressor of the galactose utilization pathway [ 39], lies within 1 kb of the mean center of the linked intervals. We therefore sequenced this gene in the four evolved strains and in the ancestor. One or two missense mutations were found in *GAL80* in all four strains ( Figure 4C). Two of the strains carry the same mutation. One of the mutations, Q392H, has been recently identified in a screen for *GAL80* mutations that cause loss of the Gal80 inhibitory activity [ 40]. Three other mutations, I361M, Q392H, and H36Y, lie in nuclear localization sequence regions of Gal80 [ 41]. The transcriptional regulation of the galactose utilization pathway, including the role of Gal80, is depicted in Figure 5A.

To test whether these mutations account for the adaptive phenotype, the endogenous *GAL80* gene of the ancestral cells was replaced with the three different mutant genes, and the growth curves of these haploid strains were compared to those of the ancestor and a *gal80Δ* strain. Compared to the ancestral allele, all the mutations in *GAL80* conferred a growth advantage during the transition from using glucose as the sole carbon source to galactose (˜2-fold increase at 6 h; Figure 5B– 5D). However, no significant growth differences were observed for cells transferred from glucose- to glucose-containing medium or from galactose- to galactose-containing medium ( Figure S7A– S7H). The ancestral strains carrying the evolved *gal80* mutations behaved similarly to the *gal80Δ* strain, suggesting that the mutations in *GAL80* cause a partial or complete loss-of-function of Gal80′s repressive activity on the galactose utilization pathway ( Figure 5E). Hence, the *gal80* mutations explain the growth advantage observed for the four evolved stains relative to the ancestor following transfer from glucose to galactose (˜1.2–1.9-fold more evolved cells than ancestral cells 6 h after transfer; Figure 5F– 5I). The slower growth observed for the ancestor compared with the evolved strains in the transition from glucose to galactose is likely the result of a growth delay while the ancestral cells repress Gal80′s inhibitory effect and induce the genes required for galactose uptake and catabolism.

We compared the effects of the mutations in *GAL80* in two strains: the evolved strains and the strains produced by transforming these mutations into the ancestral strain. Ancestral cells carrying only the evolved *gal80* mutations have a larger growth advantage relative to the ancestral *GAL80* strains than do the evolved strains from which these mutations were rescued (compare Figure 5B– 5D to 5F–5H). This difference is probably due to one or more other mutations that accumulated in the evolved strains and have a fitness cost under the conditions of our experiment. The slight decrease in fitness observed for the evolved strains (Ev2 and Ev42) relative to the ancestor when transferred from one glucose-containing medium to another supports this hypothesis ( Figure S7E– S7L).

During each cycle of the evolution experiment, diploid evolving cells were transferred from glucose- to galactose-containing medium ( Figure 4A). This selection prompted us to ask whether a single copy of the *gal80* mutations conferred any advantage on a diploid background. We compared the growth curve of ancestral diploids heterozygous for one of the three sets of *gal80* mutations to that of an ancestral diploid, a *gal80Δ*/ *gal80Δ* diploid *,* and a *GAL80*/ *gal80Δ* diploid, following transfer from glucose to galactose ( Figure 5J– 5M). The intermediate growth levels of the heterozygous *GAL80*/ *Ev-gal80* diploid and the *GAL80/gal80Δ* diploid compared with the ancestor and the *gal80Δ/gal80Δ* diploid show that all three sets of *gal80* mutations, similar to *gal80Δ,* are phenotypically semidominant. In a molecular sense, this argues that the *GAL80* gene is weakly haploinsufficient, most likely because of its need to titrate Gal4, the transcriptional activator. In an evolutionary sense, our observations suggest that the *gal80* mutations could have been initially selected for when only one copy of the mutation/s was present in the diploid.

## Discussion

We present an optimized method that maps adaptive mutations in yeast with higher precision and less work than previous linkage-based mapping methods [ 22, 24, 25]. One advantage of our method is the capacity to predict where the linked locus is most likely to lie within a mapped region, which helps prioritize sequencing and candidate gene testing. Therefore, even though our estimated 95% confidence intervals (20–88 kb [8–35 cM]) are comparable with mapping intervals identified in previous SFP-based methods that analyze ˜20 segregants individually in yeast (8–72 kb [3–29 cM]) [ 22, 24, 25], the centers of our mapping predictions are typically much closer to the actual mutations. By pooling at least 10,000 segregants from a single cross, we obtained mapping deviations of test genes that ranged from 0.2–24 kb (0.1–10 cM). In four independently evolved populations, mutations in the same gene *(GAL80)* were mapped with a mean mapping deviation of 5.7 kb, and the average position of the four predicted positions was 1 kb from the center of the *GAL80* gene. Furthermore, pooling makes mapping easier and cheaper than analyzing single segregants. We can analyze more than 10 ^7^ selected segregants simultaneously using fewer arrays (a minimum of four) than are needed to individually analyze ˜20 single segregants (20 arrays). In both methods, additional arrays (at least two) are needed for the initial, one-time prediction of SFPs. If necessary, our method can be applied to pools that contain as few as 100 segregants. This is important for organisms that produce few progeny, or for phenotypes that must be assessed by assaying individually selected segregants, which are then assembled into pools, rather than directly selecting on pooled meiotic progeny.

Since our method can simultaneously map multiple genes with high efficiency, including genes lying on the same arm *(HYG ^R^* and *NAT ^R^)* and genes affecting a quantitative phenotype *(GAL80),* our method could be useful for multigenic or QTL mapping. This combination of high-throughput genotyping with oligonucleotide arrays [ 22] and pooling [ 27] has also been applied in plants [ 33, 34], and should accelerate QTL detection compared with traditional single-segregant mapping methods in a wide variety of organisms [ 20, 21, 42, 43]. Our method has advantages and disadvantages compared to other forms of QTL mapping. We do not make assumptions on the number of contributing QTLs or the type of interactions between them, as multiple QTL and composite interval mapping methods must do [ 21]. By selecting and genotyping pools with extreme phenotypic values, we gain mapping power, but we cannot estimate the relative effect of individual QTLs on a trait. Previous studies show that QTL effect can be estimated by genotyping pools with broader phenotypic values from the lower and upper tails of the phenotypic distribution and associating the differences in phenotypic means of the two pools to differences in their marker allele frequencies [ 35, 44, 45]. Another issue is that pools lack information on the phase between genetic markers (e.g., haplotypes) and QTLs, making it hard to learn about the type of interactions between QTLs (e.g., additive or epistatic) or to recognize distinct subsets of QTLs that can independently give rise to the same trait [ 27]. Since pool genotyping is commonly used in human association studies [ 27, 30], it would be interesting to explore whether our method and its statistical framework could be extended to such studies [ 46].

We developed a computer model that simulates the mapping process to better understand the effects of various factors on mapping precision, and to improve our experimental protocols. The parameters of the model can be adjusted so that the simulations can be applied to other experimental designs, such as backcrosses, and to different organisms. Our simulations suggest that marker density and recombination rate are the major factors affecting mapping precision. While we have generated a very dense genetic map of about 10,300 DNA markers (on average ˜1 SFP/kb), the model predicts that with tighter genetic marker spacing (two to four markers per kb) our method could reach even higher mapping resolutions, corresponding to a few genes in yeast (˜1–2 cM). Tiling arrays that contain oligonucleotides that cover the whole genome and that are available for some organisms (recently including yeast [ 47]) will provide such high SFP coverage. Alternatively, different reference strains with different polymorphism distributions compared with the target strain can be used to increase genome coverage and marker density.

We showed that four independently evolved strains found the same genetic solution to repeated transitions from glucose- to galactose-containing medium and two of the strains independently acquired the same mutation. All three sets of mutations in *GAL80* reduced its ability to repress genes involved in galactose metabolism. Thus, we observed parallel evolution at the genetic level, as has been seen in viruses, bacteria, and yeast that have been experimentally adapted to stressful conditions [ 1, 2, 4], and in fish with pelvic and armor plate reduction, and albinism [ 17, 48, 49]. Mutations in *GAL3* or *GAL4* have been shown to lead to constitutive expression of the galactose utilization pathway [ 50, 51]. We did not find gain-of-function mutations in these genes, most likely because the target size for loss-of-function mutations in *GAL80* is much larger than that for gain-of-function mutations in *GAL3* or *GAL4.* All the missense mutations we found in *GAL80* lie in residues that are highly conserved across yeast species from Saccharomyces cerevisiae to Kluyveromyces lactis ( Figure S8). Our results, together with other studies [ 2, 17], support the notion that mutations in regulatory genes may lead to large benefits in populations subjected to changing environments.

Although much effort has gone into studying evolutionary changes in experimental and natural populations [ 3, 52], many questions remain. Is there correlation between the number, effect, and nature of the adaptive mutations and the molecular pathways that are subjected to the selective pressure? To what extent do evolutionary paths overlap at the genetic level between populations subjected to identical selective pressures and how does such overlap depend on the underlying network? The high-throughput and high-resolution aspects of our mapping method (freely available at http://www.cgr.harvard.edu/MutationMapping) make it amenable for such large-scale studies in yeast or higher eukaryotic organisms, as well as for studying the genetic basis of quantitative traits.

## Materials and Methods

### Yeast strains, techniques, and media.

The genotypes of the yeast strains used in this study are listed in Table S4. The target strain for our test mapping (AVS4) was a W303 strain, JYL13 *(MATa ura3–1 his3–11,15 trp1–1 ade2–1 can1–100)* transformed with three drug-resistance genes, *KAN ^R^, HYG ^R^,* and *NAT ^R^,* which had been integrated at the following intergenic locations: *KAN ^R^* on Chromosome 7 at position 413,409 bp (between *ALG13* and *RIM8*), *HYG ^R^* on Chromosome 15 at position 619,115 bp (between *YOR152c* and *PDR5*), and *NAT ^R^* at Chromosome 15 at position 960,610 bp (between *RPA43* and *RPA190*). The reference strain used for the test case is a derivative of SK1, JYL394 *(MATα ura3 (ΔSmaI-PstI) trp1::hisG leu2::hisG lys5Δ::3xHA ho::hisG GAL3)* [ 53]. The YJM789 strain was provided to us by the Ron Davis lab *(MATα ho::hisG gal2 lys2)* [ 54], and the S288c strain was BY4742 (Invitrogen, Carlsbad, California, United States). The evolved strains originated from strains isogenic with W303 (JYL243 and JYL246). Yeast transformations were carried out by the lithium acetate procedure [ 55]. Media, microbial, and genetic techniques were as described [ 56].

To map known genes, the target and reference strains were mated and single, manually isolated diploids were grown overnight in YEP (yeast extract peptone) + 2% potassium acetate (KAc) and then sporulated in 2% KAc for 3 d. Haploid segregants from this cross were grown overnight in one of three liquid media: (1) synthetic medium lacking arginine, lysine, and containing canavanine (complete synthetic media [CSM] − lysine − arginine + 60 μg/ml L-canavanine [Sigma, St. Louis, Missouri, United States]) to select for Lys ^+^, Can ^r^ cells; (2) rich medium containing geneticin, hygromycin, and nourseothricin (YPD [1% yeast extract, 2% peptone, 2% dextrose] + 400 μg/ml geneticin [G418; Gibco-BRL, Carlsbad, California, United States] + 300 μg/ml hygromycin B [Roche, Indianapolis, Indiana, United States] + 100 μg/ml nourseothricin [Clonat; Werner BioAgents, Jena, Germany]) to select for Kan ^r^, Hyg ^r^, Nat ^r^ cells; or (3) rich medium (YPD) to produce the control pool. To select for 100 segregants, the asci of the sporulated hybrid diploid were digested and the spores were immediately plated on selective medium. Colonies (100) representing individual segregants were picked and mixed in equal amounts.

### Evolution experiment.

The four evolved populations originated from the same ancestral strain in four separate, replicate experiments. In each cycle of evolution, haploid cells were grown in glucose-containing media for 4 d, mated on YPD plates and transferred to galactose-containing media for 2 d, and then put through a sporulation cycle. There were 36 such cycles for each population. The primary motivation of this experiment was to evolve mating discrimination (described in [ 37]). The evolving and ancestral cells were genetically designed so that cycles of exposure to glucose and galactose would contribute to the selection for an altered mating preference in the evolving population. During the whole procedure, the effective population size was maintained at more than 10 ^5^ cells.

### Selection for segregants expressing adaptive phenotype.

To map the adaptive mutations in the evolved strains, a single representative clone was isolated from each evolved population (see [ 37]). Since high *GAL3* expression was found in the evolved strains relative to the ancestor (unpublished results), we chose *GAL3* as a reporter gene for the adaptive glucose-galactose phenotype. The gene encoding green fluorescent protein, *GFP,* was fused to the C-terminus of *GAL3* at *GAL3*'s endogenous chromosomal location. The evolved clones and an ancestral clone (as a control) were then mated with a reference SK1 strain (JYL631 and JYL632) and sporulated. Haploid segregants in mid-log growth phase in YPD were sorted by a fluorescence-activated cell sorter (FACS; DakoCytomation MoFlo Cell Sorter, Carpinteria, California, United States) according to Gal3-GFP intensity (using excitation at 488 nm and a 505–555-nm emission filter). The brightest 5% of cells were collected and a total of 10 ^4^ cells were isolated and amplified for genomic DNA extraction. A 1:1 mixture of ancestral cells and ancestral cells transformed with the *gal80* mutations, both carrying Gal3-GFP, showed a bimodal distribution of GFP expression, demonstrating that the ancestral and evolved populations could be cleanly separated.

### Genomic DNA hybridizations.

For genotyping and linkage analysis, genomic DNA was hybridized onto Affymetrix Yeast S98 arrays ( http://www.affymetrix.com/index.affx) that contain 25mer probes designed using the genomic DNA sequence of the S288c strain. Total genomic DNA was extracted using the Qiagen Genomic-Tip 100/G kit (Qiagen, Valencia, California, United States) and brought to a final concentration of 1 μg/μl in distilled water. For each sample, 10 μg of DNA was digested, labeled, and hybridized onto an array according to standard Affymetrix protocols for RNA hybridization ( http://www.affymetrix.com/support/index.affx). We reduced the concentration of NaCl in the hybridization solution to 0.45 M in order to shift the average hybridization intensity away from saturation to an average of 1,000–3,000 U. Hybridized arrays were scanned using the Affymetrix scanner (GeneChip Scanner 3000) and the .CEL files (version 3) were used for the mapping analyses. The genomic DNA of the target and reference strains was hybridized onto eight array replicates each, and the selected and control pools onto two to four replicates.

### Array feature sequence analysis.

We refer to the oligonucleotides on microarrays as features. Each of the 136,934 Perfect Match (PM) 25mer probes on the Affymetrix Yeast Genome S98 array were blasted against the S288c genome ( ftp://genome-ftp.stanford.edu/pub/yeast/sequence/genomic_sequence/chromosomes/fasta) from which the probe sequences were designed. Only features that met the following criteria were used in this study: (1) they have a unique perfect match against the S288c genome; (2) they do not lie in repetitive regions, such as telomeres, centromeres, yeast autonomous replication sequences (ARS), or mobile genetic elements (feature positions downloaded from ftp://genome-ftp.stanford.edu/pub/yeast/sequence/genomic_sequence/other_features/other_features_genomic.fasta.gz); and (3) they do not lie in mitochondrial sequences. In cases where two fully complementary probes were found, only one of the two was kept to avoid probe redundancy. A table with the chromosome locations, *x* and *y* array coordinates, and the sequences of the resulting 120,050 probes is available at http://www.cgr.harvard.edu/MutationMapping.

### Array preprocessing and normalization.

The raw signal intensities of the PM and Mismatch (MM) features on Affymetrix yeast arrays were extracted from .CEL files (version 3) obtained from array scans. The intensities of the 120,050 features described above were read into a matrix with their array coordinates. All arrays were processed as follows: (1) Probe preprocessing. MM intensities were subtracted from their corresponding PM values [ 57], as this yielded comparable or slightly better mapping results compared with other probe preprocessing methods (see Tables S1 and S2). Negative values were set to one. (2) Normalization. To normalize between arrays and to correct for spatial hybridization inhomogeneities on the array, the intensity of each feature following MM subtraction was divided by a spatial local median intensity. A local median was calculated for each feature as the median intensity of an invariant set of PM features (nonpolymorphic between the target and reference strains) that fell within a window of 30 by 30 features centered around the given feature. Nonpolymorphic features were identified with a one-tailed two-sample *t* test at a *p* value range of 0.05–0.95. The analysis was implemented in Perl and can be downloaded at http://www.cgr.harvard.edu/MutationMapping. The user has the option of choosing between different preprocessing and normalization methods in the mapping software.

### SFP identification.

The SFPs considered in this study are array features whose mean hybridization intensities are significantly higher in the target strain relative to the reference strain. SFPs were identified using a one-tailed two-sample *t* test [ 58] between eight hybridization replicates of the target strain, *W303,* and the reference strain, SK1 at *p* = 10 ^−6^. The test statistic *t* with ν degrees of freedom for sample *b* compared with sample *a* at SFP number *i (t _i,a,b_)* is given in Equation 1 (for ν equation see [ 58], p. 129):



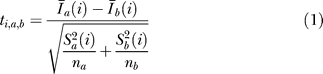



where *a* and *b* refer to the target strain and reference strain, respectively, 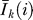 denotes the mean intensity of sample *k* onto SFP number *i* across *n _k_* replicate arrays, and 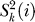 denotes the variance between *n _k_* replicate hybridization intensities for sample *k* at SFP *i*. The hybridization intensities are assumed to be normally distributed, a reasonable approximation according to our tests (unpublished data). Equal variances are not assumed for the two samples, as SFPs by definition refer to features with distinct intensities between two samples; variances were calculated from the observed replicate intensities.

### SFP verification and optimization.

To optimize our algorithm for identifying SFPs and to evaluate the specificity and sensitivity of our method, we used two strains whose genomes have been sequenced, S288c and YJM789 (sequenced by the Stanford Genome Technology Center [ 54]). We blasted the 120,050 Affymetrix probes, whose sequences were taken from S288c, against the YJM789 genome. We found that 107,448 probes are perfectly matched to at least one region in both genomes, and 12,602 probes are polymorphic (SFPs), with a prefect match to a unique sequence in S288c and no perfect match in YJM789. We hybridized the genomic DNA of S288c and YJM789 onto eight arrays each and SFPs were predicted using the array preprocessing and normalization, and SFP identification algorithms described above. The false-positive rate calculated for S288c/YJM789 can be used to estimate an FDR between any two strains at a given significance level and given number of array replicates. For this, an estimated number of false-positive SFPs between the two strains, calculated as the S288c/YJM789 false-positive rate times the estimated number of nonpolymorphic features between the two given strains, is divided by the total number of features scored as polymorphic.

### Linkage analysis of a single pool of segregants.

The linkage analysis algorithms were implemented in Matlab, and are available at http://www.cgr.harvard.edu/MutationMapping. A linkage likelihood ratio *LLR(i),* which represents the level of linkage of the *i ^th^* SFP to the selected trait, was calculated for each of the 10,330 W303/SK1 SFPs along the genome ( Equation 2). *LLR(i)* is the ratio between the probability that an SFP indexed *i* is linked to the target locus and the probability that it is unlinked:



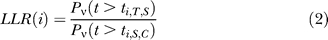



where *T* denotes the target strain, *S* the selected pool, and *C* the control pool. 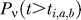 is the probability of observing *t* > *t _i,a,b_* in the *t* test for SFP *i* between sample *a* and sample *b* at ν degrees of freedom (see Equation 1), computed as



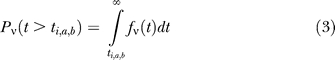



where *f*_ν_( *t*) is the *t* probability density with ν degrees of freedom. The working hypothesis is that as the distance between the SFP and the target locus decreases, the mean intensity of the selected pool onto the linked SFP increases and becomes more similar to the mean intensity of the target strain than that of the control pool. For unlinked SFPs, the intensity of the selected pool should be similar to that of the control pool.

Based on the LLR values for all SFPs, we computed an LMS at equally spaced positions along the chromosome. These positions lie on a grid defined by an offset *x _0_* (the position of the *q ^th^* SFP from the left-hand end of the chromosome) and an interval *λ* (in kilobases) (i.e., *x(j) = λ · j + x _0_* [for *j =1,2,3,…j _final_*]). *j _final_* is the index on the grid that corresponds to the *q ^th^* SFP from the right-hand end of the chromosome. At each grid point *x(j), LMS(j)* is defined as the geometric mean of the LLR scores of the *q* first SFPs to the left and *q* SFPs to the right of *x(j)* (i.e., a moving window containing *2q* SFPs, indicated here as Ω _*q*_( *j*)):



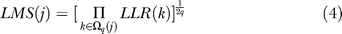



For all our linkage analyses, *LMS(j)* was plotted as a function of *x(j).* Here we used *λ* = 1 kb, and *q* = 25. This moving-window size of 50 SFPs ( *q* = 25) was selected based on our simulations (described below), as this choice gave the lower mapping deviations among the window-size range tested (10–50 SFPs; an upper limit was set due to several considerations, including chromosome length and maintaining the ability to map multiple adjacent loci; see Figure S9). Peaks whose predicted centers lay within 50 kb from a telomere were resmoothed with a window of 36 SFPs, which we found to be optimal for telomeric regions using simulations. Our simulation model can be used to find the optimal range of smoothing window size for different experimental designs, such as backcrosses, or other organisms that display different recombination rates.

### Identifying significant peaks.

The significant peaks, representing the linkage regions, were identified at a 99% confidence level using permutation analysis [ 59]. Briefly, the chromosome positions of all SFPs were randomly assigned to the observed hybridization intensities of the selected and control pools, while the coordinates of the target strain intensities remained in place. No shuffling was done between replicate array intensities or between the intensities of the selected and control pools. For each shuffling, the LMS was calculated across the entire genome and the maximum LMS value was recorded. This process was repeated 1,000 times, and the maximum LMS values from each shuffling were ranked. The 99th percentile of the ranked values was taken as the significant peak threshold.

### Estimating significant peak center.

The boundaries of each significant peak were determined using an LMS cutoff that equals 10% of the maximum height of the given peak. The center of a significant peak is defined as the position midway between two SFPs that best divide the area under the peak into two equal halves or into two areas that are closest to an equal split of the peak area. The peak area is calculated as the area under the peak between its two boundaries. To avoid false-positive spikes, peaks that were narrower than 25 kb were discarded. A 95% confidence interval around the predicted peak's center was estimated for each peak using computer simulations as described below.

### Mapping simulations and 95% confidence intervals.

We developed a computer model that simulates the entire linkage mapping process of a single selected locus. The linkage disequilibrium–based model assumes that recombination rate is proportional to the distance between two loci, and that two loci that are more than 50 cM apart are unlinked. The simulations are done at the level of the array hybridization intensities. The replicate hybridization intensities of the target strain and control pool, *I*_*a*_( *i*) *,* onto an SFP indexed *i* are sampled from a normal distribution with a mean intensity, *μ _a_*( *i*) and standard deviation, *σ _a_*( *i*) *.* These values are taken from observed intensity data of sample *a* at SFP *i* ( Equation 6).



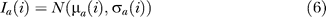



The mean intensity of the selected pool onto SFP *i* is calculated according to Equation 7:



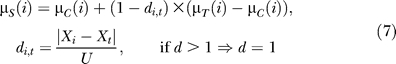



where *d _i,t_* is the relative genetic distance between an SFP at position *X _i_* and a simulated target locus at position *X _t_,* and *U* denotes the average unlinked physical distance. We assume *d _i,t_* is proportional to the recombination rate between two loci, and *U* = 125 kb (˜50 cM) based on an average recombination rate of 1 cM per 2.5 kb in yeast. A relative distance ( *d _i,t_*) of 1 represents no linkage, while a relative distance of 0 represents 100% linkage. *T* represents the target strain, *C* the control pool, and *S* the selected pool. The standard deviation of the hybridization intensities of the selected pool onto SFP *i* is derived from the coefficient of variation (μ/σ) of the control pool, as shown in Equation 8:



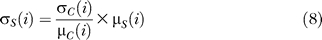



Finally, the replicate hybridization intensities of the selected pool onto SFP *i* are sampled from a normal distribution with a mean intensity, *μ _S_*( *i*) *,* and a standard deviation, *σ _S_*( *i*) ( Equation 6). The linkage analysis procedure described in the previous section is applied to the simulated hybridization intensities of replicate arrays. The absolute value of the distance between the estimated center of the detected significant peak and the actual position of the simulated target locus is recorded for each simulation. The mapping deviation is defined as the 95th percentile of the ranked deviations calculated from *n* simulation runs. In this work, *n* = 1,000.

### Testing the effect of different factors on mapping precision.

We used simulations to test the effect of various factors on mapping precision of a target locus positioned in the middle of an average-length yeast chromosome (700 kb; Figure 3). Five repeats of n = 1,000 simulation runs were done for each set of parameters tested. Aside from the varying factor, parameters were chosen according to those used or observed in our mapping test case, including an average recombination rate of 1 cM per 2.5 kb, SFP FDR of 6%, mean SFP density of 0.91 SFP/kb, four array replicates for the selected and control pools and eight replicates for the target strain, smoothing window size of 50 SFPs, and the SFP mean intensities and standard deviations of the target strain and control pool were randomly sampled without replacement from the distribution of replicate SFP intensities from our test case experiments. For each simulation run, a different subset of SFPs was randomly flagged as false positive, and the intensities of the false SFPs were randomly sampled from the observed intensities of the target strain at nonpolymorphic features.

### 95% confidence interval estimation.

To estimate the 95% confidence intervals for a predicted linked region, a simulated target locus was positioned at the predicted center, and the 95th percentile of the ranked mapping deviations from 1,000 simulation runs was recorded. The distribution of SFP positions observed in our mapping experiments for a given chromosome was used for the simulations, as well as the observed mean intensities and coefficients of variation of the target strain and control pool at the corresponding SFPs. The estimated 95% confidence intervals are rough estimates, as a uniform mean recombination rate is assumed across the whole genome due to lack of detailed data on the local variation in recombination rate. As a result, when local recombination rates are higher than average, the 95% confidence intervals are placed too far away from the predicted position of the target locus, and when local rates are lower than average the intervals are placed too close to the predicted position.

### *GAL80* sequencing.

To sequence *GAL80,* we amplified a fragment on Chromosome 13 from position 171,100 to 173,315 bp. We designed sequencing primers ˜400 bp apart on the same strand with ˜200-bp separations between primers on opposite strands. Both strands of the PCR product were sequenced using a Big Dye Terminator cycle sequencing kit (Applied Biosystems, Foster City, California, United States) and the product read with an ABI3100 Genetic Analyzer. The sequence readouts were assembled into a single contig using ContigExpress (part of VectorNTI software; Invitrogen); 2–4 × coverage was obtained.

### Mutation reconstruction and growth curve assays.

To reconstruct the evolved mutations in the ancestral strain, the mutant *gal80* genes and flanking sequences were amplified by PCR from the evolved strains and transformed into an ancestral strain whose *GAL80* gene was replaced by a *URA3* gene. The transformants were then plated on 5-fluoro-orotic acid–containing medium which selects against the *URA3* gene and thus for cells where *gal80Δ::URA3* has been replaced by the mutant *gal80* genes. The structure of the *GAL80* locus in each of the transformants was checked by PCR, and the genomic DNA was sequenced to show that the mutant allele had been properly integrated without introducing any further mutations. For growth curves, cells were grown in YPD (2% glucose) or YEP + 2% galactose (in the galactose-to-galactose transfers) overnight, diluted and refreshed in the same medium for 4 hours and then transferred to YPD or YEP + galactose medium. Cell numbers were estimated from the optical densities of the cell cultures using a spectrophotometer (DU640B; Beckman Coulter, Marseille, France). In each assay, at least three independent cultures were set up and their average and standard deviation is shown at each sample point.

## Supporting Information

Figure S1 Sensitivity and Specificity of SFP Identification Evaluated Using Two Yeast Strains with Known Sequences (A) The true-positive, false-positive, and false discovery rates of S288c/YJM789 SFP identification are presented in percentages for a wide range of *p* value cutoffs (10 ^−8^ to 0.1). True-positive rate refers to the fraction of features that are truly polymorphic that are scored as polymorphic; false-positive rate refers to the fraction of nonpolymorphic features that are scored as polymorphic; and FDR refers to the fraction of features that are scored as polymorphic that are not truly polymorphic. The true S288c/YJM789 SFPs were determined by blasting the Affymetrix probe sequences that were derived from the S288c genome, against the YJM789 genome. Of the 120,050 Affymetrix probes tested, 107,448 were found to be nonpolymorphic between the strains, and 12,602 were found to be polymorphic (SFPs). The rates presented here were calculated using a one-tailed two-sample *t* test between eight replicate arrays for each strain. When considering only the SFPs whose polymorphisms lie in the central 15 bp of the 25mer probe (7,588 SFPs), the true-positive rate of SFP identification increased by 15%–30% compared with using all SFPs (third column). The true-positive rate is plotted as a function of false-positive rate (B) and FDR (C) in percentages over a *p* value cutoff range of 10 ^−8^ to 0.1. The dots correspond to the *p* values given in (A) from *p* = 10 ^−8^, the far left point, to *p* = 0.1, the far right point. Of the *p* value cutoffs in (A), *p* = 10 ^−6^ gave the maximum true-positive–to–false discovery rate ratio (labeled in red in panels [A], [B], and [C]). We used the FDR and not the false-positive rate to evaluate the specificity of our SFP identification, as the fraction of SFPs that are false is more relevant for mapping.
(210 KB PDF)

Figure S2 Distribution of the Distances between Consecutive W303/SK1 SFPs along the Genome At a *p* value cutoff of 10 ^−6^, 10,330 W303/SK1 SFPs were identified using a one-tailed two-sample *t* test on eight replicate arrays for each strain.
(A) The percentage of SFP pairs is plotted as a function of the distance between consecutive SFPs along all 16 chromosomes in kb units, in 1-kb bins centered around the bin points. Note the logarithmic scale of the *y*-axis. The mean SFP spacing is 1.14 kb (0.5 cM).
(B) A cumulative distribution of the percentage of SFP pairs that are less than X kb apart is plotted as a function of the distance between all consecutive SFPs (X). About 73% of the SFP pairs are less than 1 kb (0.4 cM) apart, and 99% of the SFP pairs are less than 11 kb (4.4 cM) apart. Note the *y*-axis scale is from 70% to 100%.
(214 KB PDF)

Figure S3 Whole-Genome Mapping of Five Test Case Genes Using Different Pool Sizes The LMS is plotted across all 16 yeast chromosomes for four selected W303/SK1 segregant pools: (A–B) 10 ^7^, (C–D) 10 ^4^, and (E–F) 10 ^2^ segregants resistant to geneticin, hygromycin, and nourseothricin *(KAN ^R^, NAT ^R^,* and *HYG ^R^),* and (G–I) 10 ^7^ segregants resistant to canavanine that are prototrophic to lysine *(can1* and *LYS5).* The five peaks that located the five selected genes all fell above the peak cutoffs estimated for each selected pool separately at 99% confidence (horizontal dashed lines drawn only in [B], [D], [F], [H], and [J]). The peaks are labeled **a** through **e** according to the gene they map: **a,** *KAN ^R^;* **b,** *NAT ^R^;* **c**- *HYG ^R^*, **d,** *can1;* and **e,** *LYS5.* See Figure 2E and Table 1 for mapping deviations of predicted peak centers from the corresponding linked genes and estimated 95% confidence intervals. (B), (D), (F), and (H) are *y*-axis close-ups of (A), (C), (E), and (G), respectively. A single false-positive peak on Chromosome 10 was detected in the pools selected for resistance to geneticin, hygromycin, and nourseothricin ([A–D]; labeled with a green asterisk). See text for possible explanation for observing this peak. The signal-to-noise levels are high and appear to slightly decrease as the segregant pools size decreases. A sliding window of 50 SFPs was used for all plots except for (I), where a smoothing window of constant chromosome size (35 kb) was used. The height of the *LYS5* peak has increased disproportionately by using a smoothing window of 35 kb (I) versus 50 SFPs (G–H). We believe this reflects the lower SFP density around *LYS5* (0.52 SFPs/kb), which is ˜1.7-fold lower than the density around *can1* (0.88 SFPs/kb) and than the genome's mean SFP density (an interval of 30 kb around the genes was used for SFP density comparison). (J) To test whether we could map genes that are not fully enriched in a selected pool of segregants, we mixed the pool of segregants selected in geneticin, hygromycin, and nourseothricin (from [A]) with the control pool at a 1:1 ratio. About 75% of the segregants in the resulting pool should carry the three drug resistance genes if the initial selection was perfect. The three largest peaks correspond to the three mapped genes though the signal is much lower than in (A) (see also Figure S5). The *x*-axis labels are the numbers of the chromosomes, which are shown to scale.
(1.1 MB PDF)

Figure S4 Whole-Genome Mapping of Five Test Case Genes Using a Different Array Preprocessing Method Whole-genome mapping plots of our five test case genes are presented using a different array preprocessing method that considers only the PM probes and not the MM ones, as does the method used in this paper (PM minus MM). The preprocessing method includes taking the logarithm on base 10 of each PM value and dividing it by the median log _10_ PM of the local invariant probes on the array, similar to an approach used in previous single segregant mapping methods in yeast [ 22, 24, 25]. The arrays were processed using this method for both the SFP identification at a *p* value cutoff of 10 ^−6^ and for the linkage analyses. The LMS is plotted across all 16 yeast chromosomes for five different selected W303/SK1 segregant pools: (A) 10 ^7^, (B) 10 ^4^, and (C) 10 ^2^ segregants resistant to geneticin, hygromycin, and nourseothricin *(KAN ^R^, NAT ^R^,* and *HYG ^R^),* (D) 10 ^7^ segregants resistant to canavanine that are prototrophic for lysine *(can1* and *LYS5),* and (E) 10 ^7^ segregants resistant to geneticin, hygromycin, and nourseothricin where only about 75% of the segregants contain the drug resistance genes (details in Figure S3J). The five peaks that located the five selected genes all fell above the estimated 99% confidence peak cutoffs in (A–D) (the cutoffs were not drawn as they are not visible on this *y*-axis scale). The mapping deviations of the mapped genes in all five panels ranged from 0.2 kb to 32 kb (0.1–13 cM), and their estimated 95% confidence interval ranged from ±5 kb to ±38 kb (±2–15 cM). The peaks are labeled **a** through **e** according to the gene they map: **a,** *KAN ^R^;* **b,** *NAT ^R^;* **c,** *HYG ^R^;* **d,** *can1;* and **e,** *LYS5.* A single false-positive peak on Chromosome 10 was found in the pools selected for resistance to geneticin, hygromycin and nourseothricin ([A,B,C,E]; labeled with a green asterisk). The significant peak cutoff is drawn in (E) (horizontal dashed line). The *x*-axis labels are the numbers of the chromosomes, which are shown to scale. The mapping method appears to be robust to the array preprocessing method used, although the PM minus MM method seems to yield slightly better mapping precisions on average than the PM only (log _10_ PM) method (see Table S1). We estimated the mapping deviations of 23 loci (including several mapping repetitions of drug resistance genes, different pool sizes, 75% enrichment of test genes, and *gal80* mutations) using both methods. PM minus MM mapped 16 of 23 loci with higher precision than log _10_ PM. Assuming the two methods are equally good, and thus each method has a probability of 0.5 of yielding a smaller mapping deviation for each measurement, the probability of seeing a bias of 16 to 7 or larger using the binomial distribution is *p* < 0.05.
(545 KB PDF)

Figure S5 Distribution of Whole-Genome LMSs following Different Enrichment Levels of Target Alleles in the Selected Pool The signal-to-noise ratio of the whole-genome LMS is high even with only 75% enrichment of the target alleles in the selected pool. The distribution of the LMS values of all datapoints along the genome is presented for the mapping of the three drug-resistance genes, *KAN ^R^, HYG ^R^,* and *NAT ^R^,* that are represented in either (A) 90%–100% (from Figure S3A) or (B) 70%–75% (from Figure S3J) of the segregants in the selected pool. The red arrows mark the 95th percentile of the ranked LMS values, and the black arrows mark the range of the peak heights of the three mapped genes. Note the *x*-axis is on a log scale and on the same scale for the two panels. Although the LMS values are much smaller with 75% enrichment, the signal-to-noise ratio is still high. With 75% enrichment, the peak heights are 33- to 39-fold larger than the 95th percentile of the ranked LMS values, while with 90%–100% enrichment, the peak heights are 18- to 190-fold larger than the 95th percentile LMS. In order not to lose true-positive peaks that fall below an estimated cutoff, peaks can be ranked according to height or area, and lower ranked peaks that fall below the cutoff can be tested later. To increase mapping sensitivity, a control pool could be made up of segregants from the opposite extreme tail of the phenotype distribution to that of the selected pool (i.e., segregants that do not express the trait of interest or that express it to a low extent) [ 45] instead of segregants randomly sampled from the phenotype distribution (as was done in this work). This should be especially useful for target loci that have an approximately additive effect. Furthermore, our simulation model can be used to estimate significant peak cutoffs given different projected levels of enrichment of the target alleles in the selected pool.
(265 KB PDF)

Figure S6 Whole-Genome Mapping of Four Evolved Strains That Independently Adapted to Glucose-to-Galactose Transition W303/SK1 segregants that expressed high levels of Gal3-GFP were selected using flow cytometry and mapped. The LMS is plotted across all 16 yeast chromosomes for each of the four evolved strains: Ev2 (A), Ev14 (B), Ev42 (C), and Ev43 (D). The horizontal dashed lines mark the significant peak cutoffs estimated for each selected pool of Evolved/SK1 segregants at 99% confidence. The peak on Chromosome 13 is strongly linked to *GAL80,* in which we subsequently found missense mutations that were confirmed experimentally to be adaptive in all four strains. The peak on Chromosome 4 found only in Ev2 coincides with the *GAL3* locus that was fused to a GFP. This peak did not appear in the other three strains, since only the initial Ev2/SK1 hybrid diploid had one copy of *GAL3-GFP* in the evolved strain's chromosome copy and not in the reference strain's copy (SK1). In the other Ev/Sk1 diploids, *GAL3-GFP* was present on both copies of Chromosome 4. A sliding window of 50 SFPs was used for all plots. The *x*-axis labels are the numbers of the chromosomes, which are shown to scale.
(474 KB PDF)

Figure S7 The Effect of *gal80* Mutations in Glucose–Glucose and Galactose–Galactose Transitions
(A–H) Ancestral cells carrying the *gal80* mutations do not display a growth advantage relative to the ancestor, when transferred between media containing the same carbon source (glucose or galactose), similar to a *GAL80* delete strain. Following transfer from galactose- to galactose-containing medium (A–D) or glucose- to glucose-containing medium (E–H), cell density (OD) was measured for ancestral haploids whose *GAL80* gene was replaced with one of three *gal80* mutant forms: A0-Ev2, A0-Ev14, and A0-Ev43; Ev42 and Ev43 have the same mutation in the coding region. For comparison, the growth curve of a *GAL80* knockout strain ([D,H]; A0- *gal80*Δ) was measured under the same conditions. All growth curves are compared to that of the ancestor (A0).
(I–L) The evolved strains, Ev14 and Ev43, display a similar growth curve to that of the ancestor (A0) when transferred from glucose- to glucose-containing medium, while Ev2 and Ev42 are slightly less fit following the transfer.
Mean cell density and a standard deviation from at least three independent cultures were plotted for each data point for (A–L). Error bars that are not visible are smaller than the point.
(255 KB PDF)

Figure S8 Sequence Comparison of Gal80 Yeast Homologues A protein alignment of the regions in Gal80 that contain the residues that were mutated in the evolved strains is presented for six yeast species. The residues where a mutation occurred are highlighted in black and the mutations are written below the alignment in red. The percentage of amino acid identity between S. cerevisiae and K. lactis is 58%. ClustalW [ 60] was used to do the multiple sequence alignment.
(208 KB PDF)

Figure S9 Mapping Precision as a Function of LMS Smoothing Window Size We tested the effect of the LMS smoothing window size on mapping precision, using simulations where target loci positioned in the middle of a representative yeast chromosome of length 700 kb were mapped. The mapping deviation is the absolute difference of the predicted target locus position from its simulated position in kb. Each datapoint is averaged over the mapping deviations of 11 different target loci positioned in 10-kb increments from positions 300 kb to 400 kb along the chromosome. For each window size the standard deviation between the mapping deviations of the eleven loci is represented with error bars. Aside from the smoothing window size variable, parameters were set according to those used or observed in our mapping test case (for more details see the Mapping Simulations section in Materials and Methods). Mapping deviation decreases as a function of smoothing window size in the range of 10–30 SFPs per window, while in the range of 30–50 SFPs, mapping deviation is at best weakly dependent on window size.
(201 KB PDF)

Protocol S1 Factors That Affect LMS (25 KB DOC)

Table S1 Mapping Precision as a Function of Different Array Preprocessing Methods We tested the effect of different array preprocessing methods on mapping precision and found that our method is fairly insensitive to the method used. The mapping deviations of the predicted positions of the five test case genes from their real centers are given in kb. The 10,330 W303-SK1 SFPs identified using the PM-MM preprocessing method at a *p* value cutoff of 10 ^−6^ were used for the comparison. The mean mapping deviations and standard deviations for *KAN ^R^, HYG ^R^,* and *NAT ^R^* were calculated from three separate mapping experiments. To calculate a local background (b1, b2, b3) we divided the array into 10 × 10 squares and subtracted the following values from each PM: (1) median of the MM values lying in the square encompassing the given PM (b1); (2) mean of the lower 5% of ranked PM and MM values in the corresponding square (b2); and (3) mean of the lower 2% of ranked PM values in the corresponding square (b3). These methods are similar to those used by the Affymetrix GeneChip software ( http://www.affymetrix.com/support/technical/whitepapers.affx; Statistical Algorithms Description Document [ 57]) and Li and Wong's dChip software [ 61]. All intensities were normalized by a median of a spatially local set of invariant PM values. For log _10_ PM the logarithm of PM was divided by the median log _10_ PM of the local invariant probes, similar to the approach used in previous single segregant mapping methods in yeast [ 22, 24, 25]. In the software of our mapping method, the user will have the option of choosing between different array preprocessing methods, including those that do not use the MM, and will be able to adjust tunable parameters, such as the percentile of ranked intensities used for background subtraction ( http://www.cgr.harvard.edu/MutationMapping). This will allow users to find the optimal method for their mapping system.
(43 KB DOC)

Table S2 The Effect of Using Different Array Preprocessing Methods for Identifying SFPs on Mapping Precision The mapping precision of our method is robust to the array preprocessing method used for calling features polymorphic (SFPs) at a *p* value cutoff of 10 ^−6^. Different probe preprocessing methods were used to identify SFPs, and the linkage analysis was then done using the PM-MM preprocessing method. This allowed us to isolate the effect of the preprocessing method used to find SFPs on mapping precision. The mapping deviations of the predicted positions of the five test case genes from their real centers are given in kilobases. Although the true-positive–to–false discovery rate ratios may vary between the different methods (unpublished data), this does not appear to have a significant effect on the final outcome of the mapping. This is in accordance with the prediction of our simulations, that false SFPs do not have a significant effect on mapping precision ( Figure 3C). The local backgrounds (b1, b3) are defined in Table S1. The mean mapping deviations and standard deviations for *KAN ^R^, HYG ^R^,* and *NAT ^R^* were calculated from three separate mapping experiments.
(36 KB DOC)

Table S3 Mapping Precision of Test Case Genes as a Function of Array Replicate Number The mapping deviations of the predicted gene locations from their actual centers are presented here as an average of the absolute deviations calculated from all possible combinations of duplicate, triplicate, and quadruplicate arrays out of four replicates from a single mapping experiment. Mapping deviation is given in kb. The number of array replicates refers to the selected pool, control pool, and the target strain, *W303. R ^2^* denotes the correlation of coefficient of mapping deviation as a function of replicate number.
(31 KB DOC)

Table S4 Genotype of Yeast Strains (33 KB DOC)

### Accession Numbers

The National Center for Biotechnology Information (NCBI) ( http://www.ncbi.nlm.nih.gov) accession numbers for the genes and gene products discussed in this paper are S288c genome (NC_001133–NC_001148) and YJM789 genome (AAFW01000001–AAFW01000150). The *Saccharomyces* Genome Database ( http://www.yeastgenome.org) accession numbers for genes and gene products discussed in this paper are *can1* (S000000789), *LYS5* (S000000789), *GAL3* (S000002416), *GAL80* (S000004515), *GAL4* (S000006169), and Gal2 (S000004071).

## Abbreviations

FDR: false discovery rate
GFP: green fluorescent protein
LLR: linkage likelihood ratio
LMS: linkage map score
MM: Mismatch
PM: Perfect Match
QTL: quantitative trait locus
SFP: single-feature polymorphism
