# Supplementary material for: High-Resolution Mutation Mapping Reveals Parallel Experimental Evolution in Yeast
Source: PLoS Biol. 2006 Jul 25;4(8):e256. doi: 10.1371/journal.pbio.0040256 (PMC1514788; doi:10.1371/journal.pbio.0040256)

**Figure S1**

**A**

| p-value cutoff | True positive rate, % | True positive rate of central 15bp, % | False positive rate, % | False discovery rate, % |
|---|---|---|---|---|
| $10^{-8}$ | 9.59 | 12.30 | 0.03 | 2.58 |
| $10^{-7}$ | 20.47 | 26.32 | 0.11 | 4.37 |
| $10^{-6}$ | 35.22 | 44.97 | 0.30 | 6.74 |
| $10^{-5}$ | 50.72 | 64.42 | 0.81 | 11.94 |
| $10^{-4}$ | 64.32 | 79.88 | 2.23 | 22.82 |
| $10^{-3}$ | 72.57 | 87.74 | 5.70 | 40.08 |
| 0.01 | 77.61 | 91.47 | 11.42 | 55.64 |
| 0.05 | 80.34 | 93.28 | 15.71 | 62.50 |
| 0.1 | 81.46 | 93.85 | 17.83 | 65.11 |

**B**

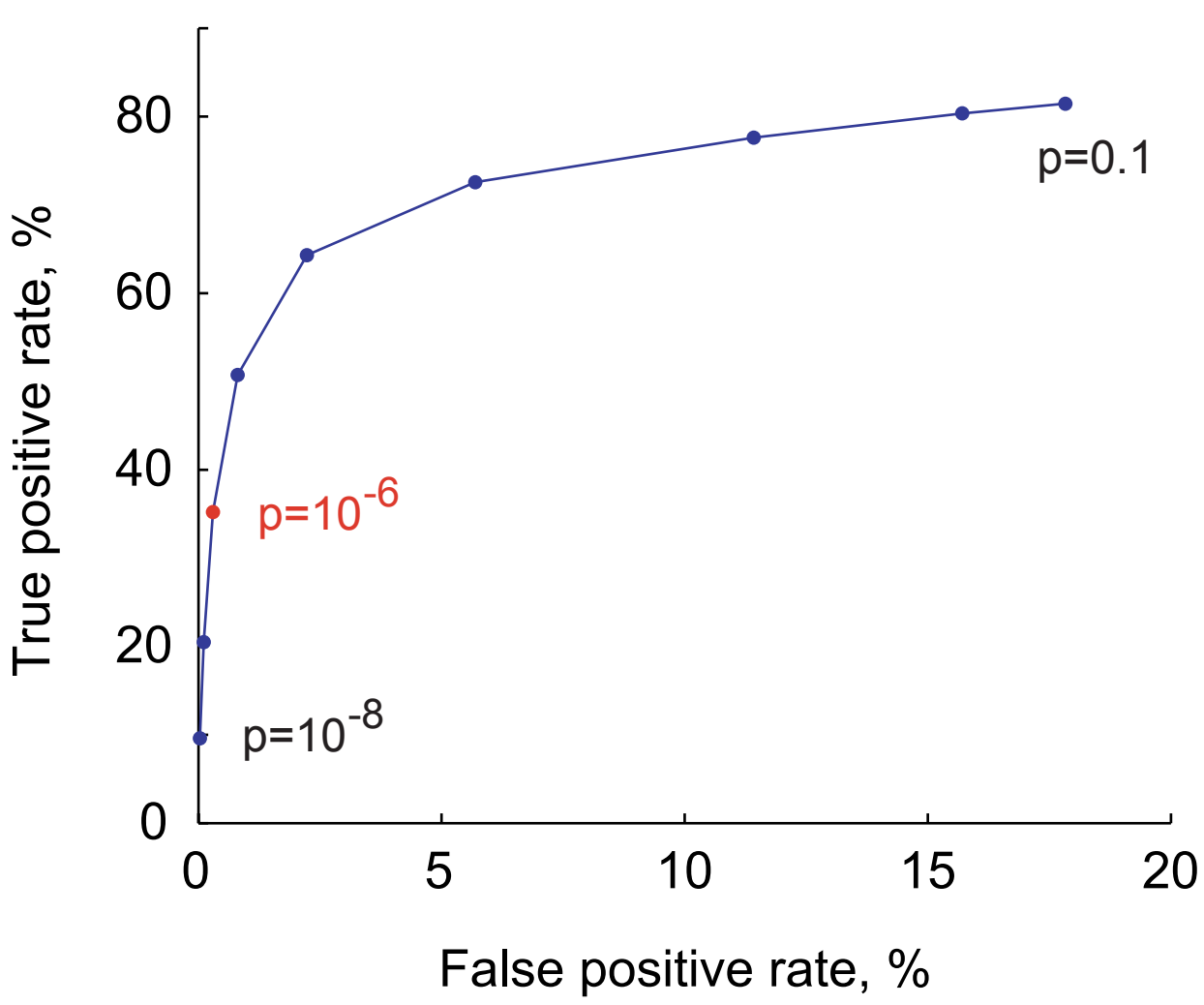


**C**

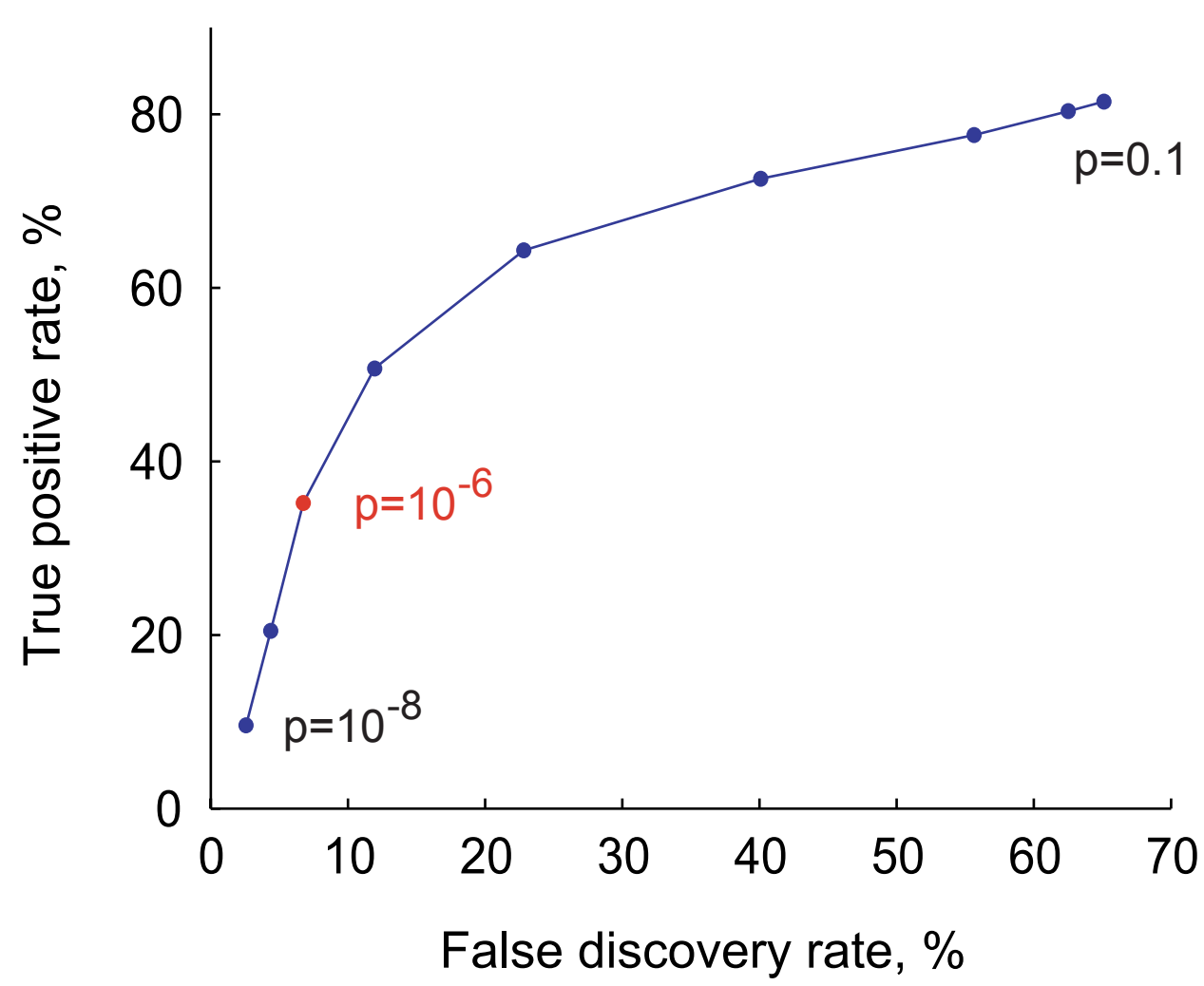

Supplement: Supplementary file 1 [file pbio.0040256.sg001.pdf]
