# Supplementary material for: High-Resolution Mutation Mapping Reveals Parallel Experimental Evolution in Yeast
Source: PLoS Biol. 2006 Jul 25;4(8):e256. doi: 10.1371/journal.pbio.0040256 (PMC1514788; doi:10.1371/journal.pbio.0040256)
Supplement: Supplementary file 2 [file pbio.0040256.sg002.pdf]

Figure S2

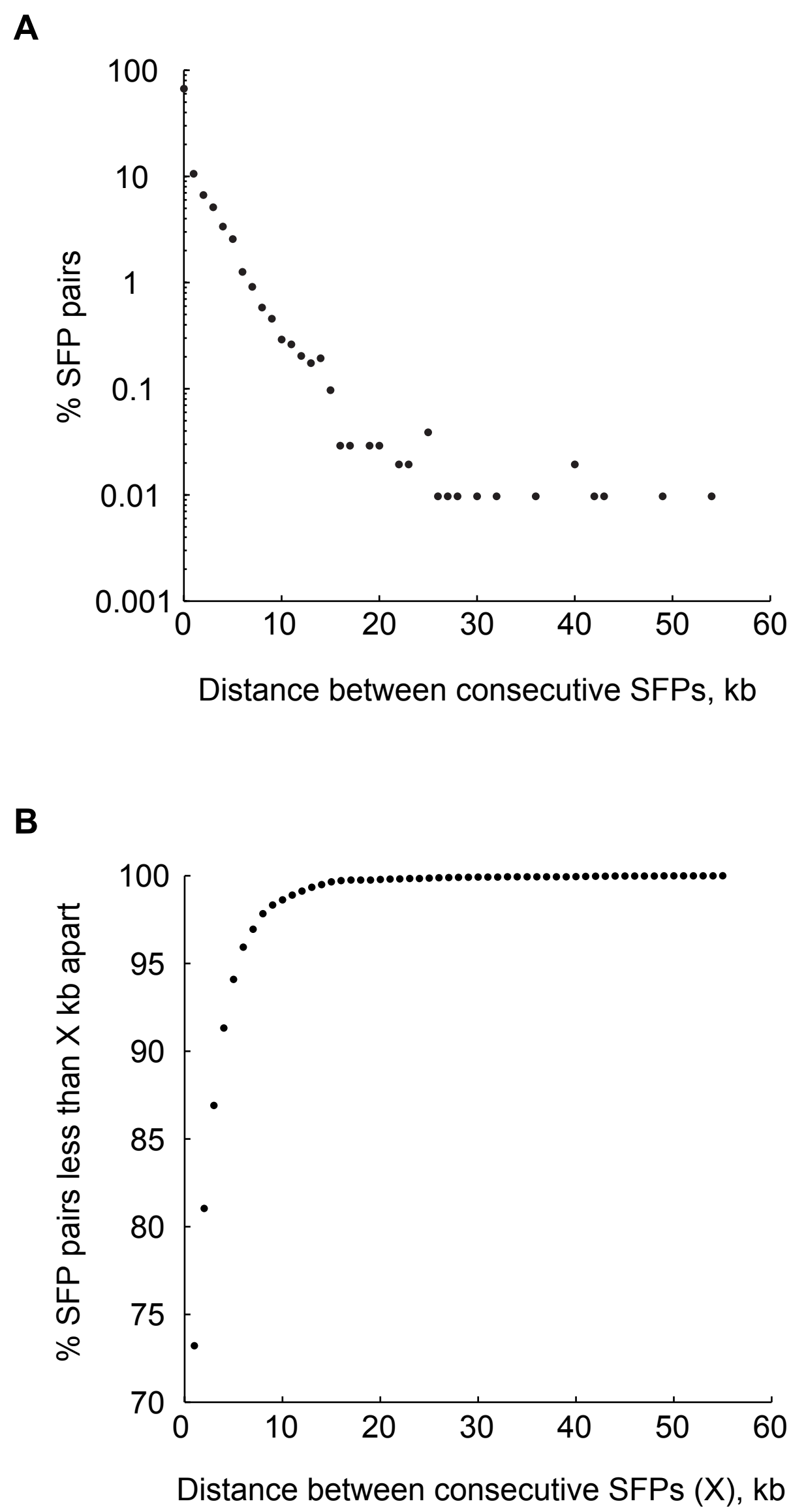
A
100
10
1
0.1
0.01
0.001
% SFP pairs
0
10
20
30
40
50
60
Distance between consecutive SFPs, kb
B
100
95
90
85
80
75
70
% SFP pairs less than X kb apart
0
10
20
30
40
50
60
Distance between consecutive SFPs (X), kb
