# Supplementary material for: High-Resolution Mutation Mapping Reveals Parallel Experimental Evolution in Yeast
Source: PLoS Biol. 2006 Jul 25;4(8):e256. doi: 10.1371/journal.pbio.0040256 (PMC1514788; doi:10.1371/journal.pbio.0040256)
Supplement: Supplementary file 3 [file pbio.0040256.sg003.pdf]

Figure S3 (A-F)

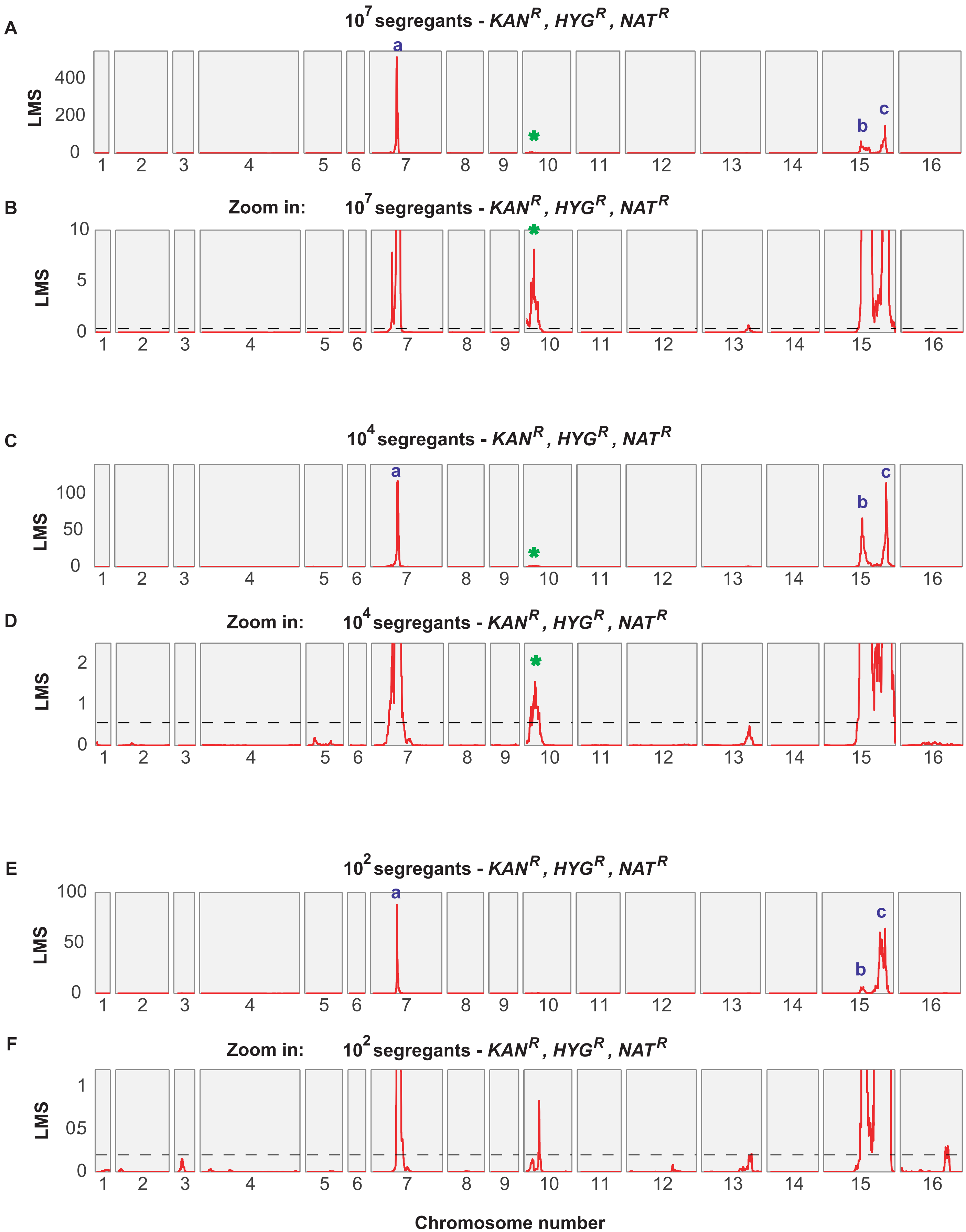
A
10^7 segregants - *KAN^R*, *HYG^R*, *NAT^R*
B
Zoom in: 10^7 segregants - *KAN^R*, *HYG^R*, *NAT^R*
C
10^4 segregants - *KAN^R*, *HYG^R*, *NAT^R*
D
Zoom in: 10^4 segregants - *KAN^R*, *HYG^R*, *NAT^R*
E
10^2 segregants - *KAN^R*, *HYG^R*, *NAT^R*
F
Zoom in: 10^2 segregants - *KAN^R*, *HYG^R*, *NAT^R*
LMS
a
b
c
1 2 3 4 5 6 7 8 9 10 11 12 13 14 15 16
Chromosome number

Figure S3 (G-J)

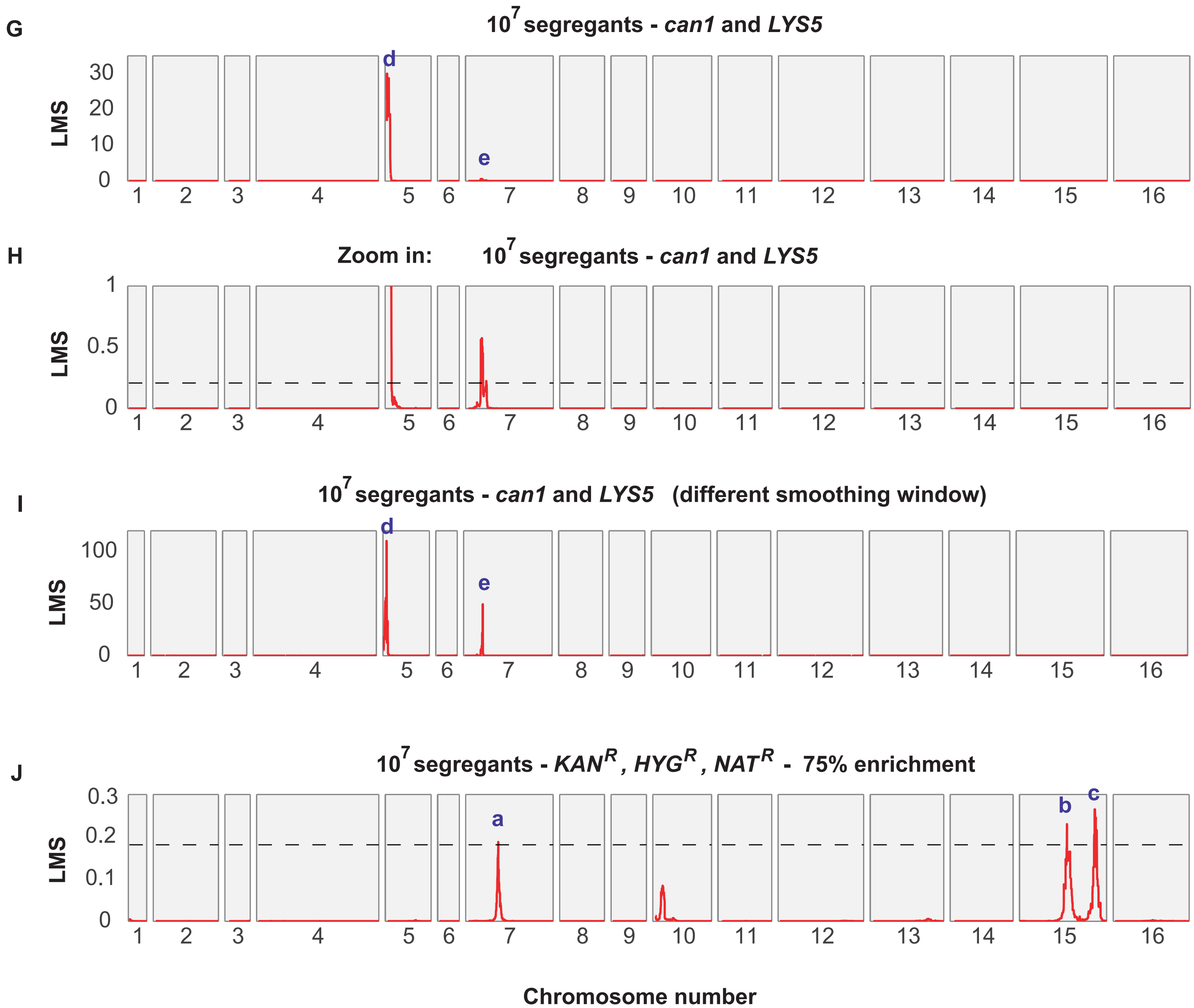
G
10^7 segregants - *can1* and *LYS5*
LMS
30
20
10
0
d
e
1 2 3 4 5 6 7 8 9 10 11 12 13 14 15 16
H
Zoom in: 10^7 segregants - *can1* and *LYS5*
LMS
1
0.5
0
1 2 3 4 5 6 7 8 9 10 11 12 13 14 15 16
I
10^7 segregants - *can1* and *LYS5* (different smoothing window)
LMS
100
50
0
d
e
1 2 3 4 5 6 7 8 9 10 11 12 13 14 15 16
J
10^7 segregants - KAN^R, HYG^R, NAT^R - 75% enrichment
LMS
0.3
0.2
0.1
0
a
b
c
1 2 3 4 5 6 7 8 9 10 11 12 13 14 15 16
Chromosome number
