# Supplementary material for: High-Resolution Mutation Mapping Reveals Parallel Experimental Evolution in Yeast
Source: PLoS Biol. 2006 Jul 25;4(8):e256. doi: 10.1371/journal.pbio.0040256 (PMC1514788; doi:10.1371/journal.pbio.0040256)
Supplement: Supplementary file 5 [file pbio.0040256.sg005.pdf]

Figure S5

A

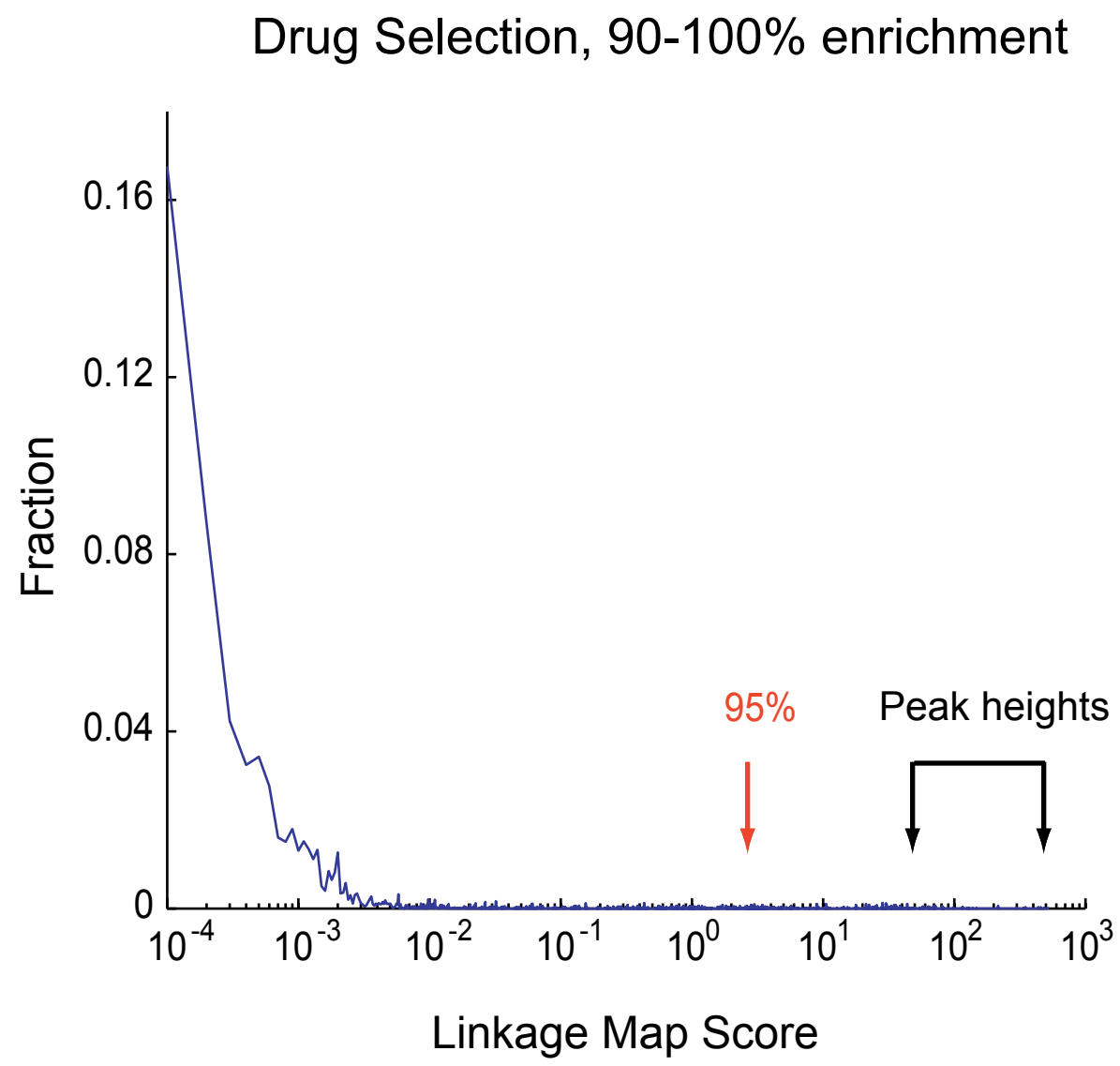
Drug Selection, 90-100% enrichment
Fraction
0.16
0.12
0.08
0.04
0
95%
Peak heights
10^-4
10^-3
10^-2
10^-1
10^0
10^1
10^2
10^3
Linkage Map Score

B

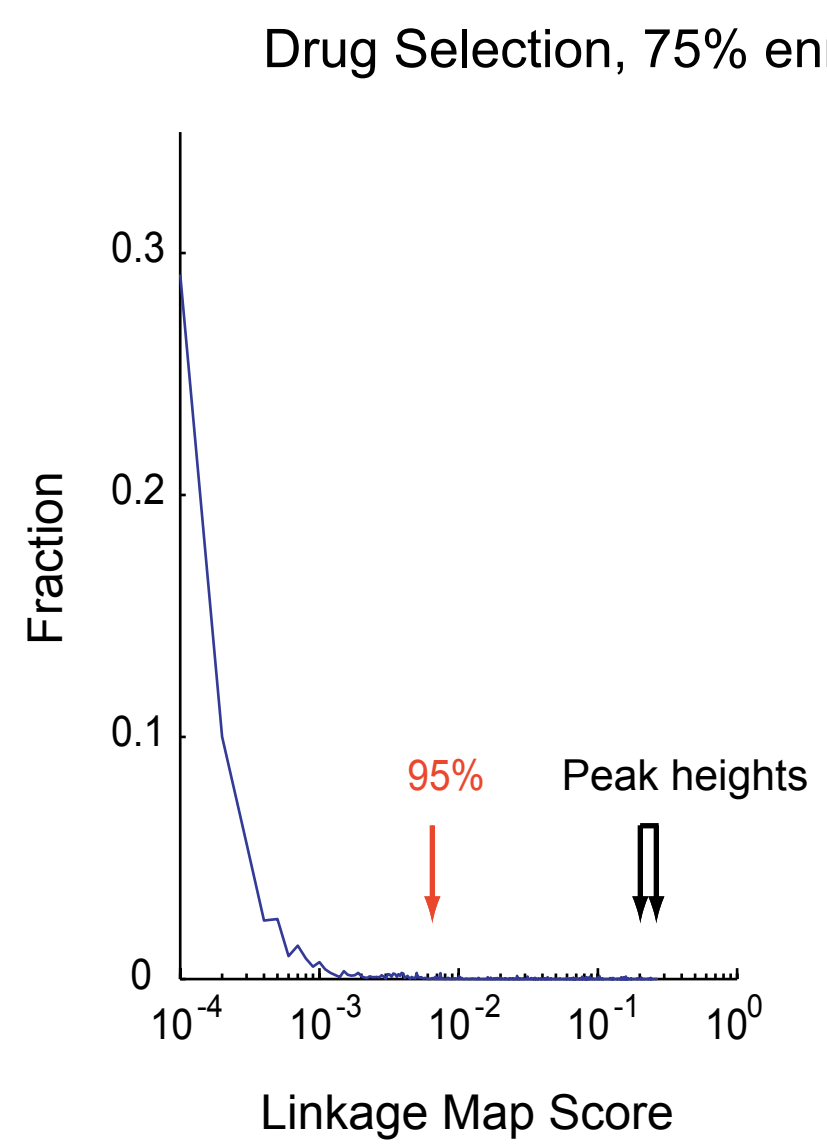
Drug Selection, 75% enrichment
Fraction
0.3
0.2
0.1
0
95%
Peak heights
10^-4
10^-3
10^-2
10^-1
10^0
Linkage Map Score
