# Supplementary material for: High-Resolution Mutation Mapping Reveals Parallel Experimental Evolution in Yeast
Source: PLoS Biol. 2006 Jul 25;4(8):e256. doi: 10.1371/journal.pbio.0040256 (PMC1514788; doi:10.1371/journal.pbio.0040256)
Supplement: Supplementary file 6 [file pbio.0040256.sg006.pdf]

Figure S6

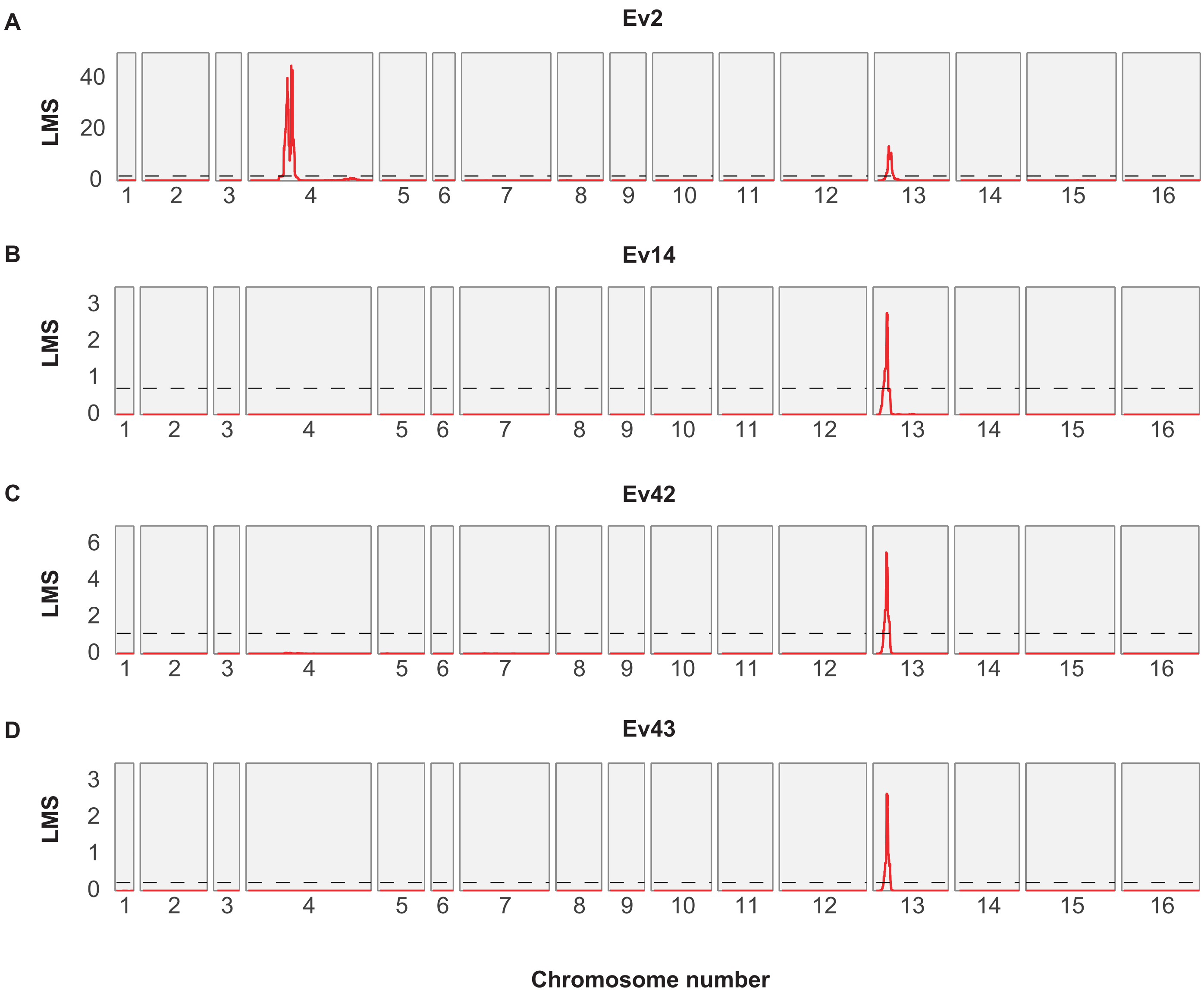
A
Ev2
LMS
40
20
0
1 2 3 4 5 6 7 8 9 10 11 12 13 14 15 16
B
Ev14
LMS
3
2
1
0
1 2 3 4 5 6 7 8 9 10 11 12 13 14 15 16
C
Ev42
LMS
6
4
2
0
1 2 3 4 5 6 7 8 9 10 11 12 13 14 15 16
D
Ev43
LMS
3
2
1
0
1 2 3 4 5 6 7 8 9 10 11 12 13 14 15 16
Chromosome number
