# Supplementary material for: High-Resolution Mutation Mapping Reveals Parallel Experimental Evolution in Yeast
Source: PLoS Biol. 2006 Jul 25;4(8):e256. doi: 10.1371/journal.pbio.0040256 (PMC1514788; doi:10.1371/journal.pbio.0040256)
Supplement: Supplementary file 7 [file pbio.0040256.sg007.pdf]

Figure S7

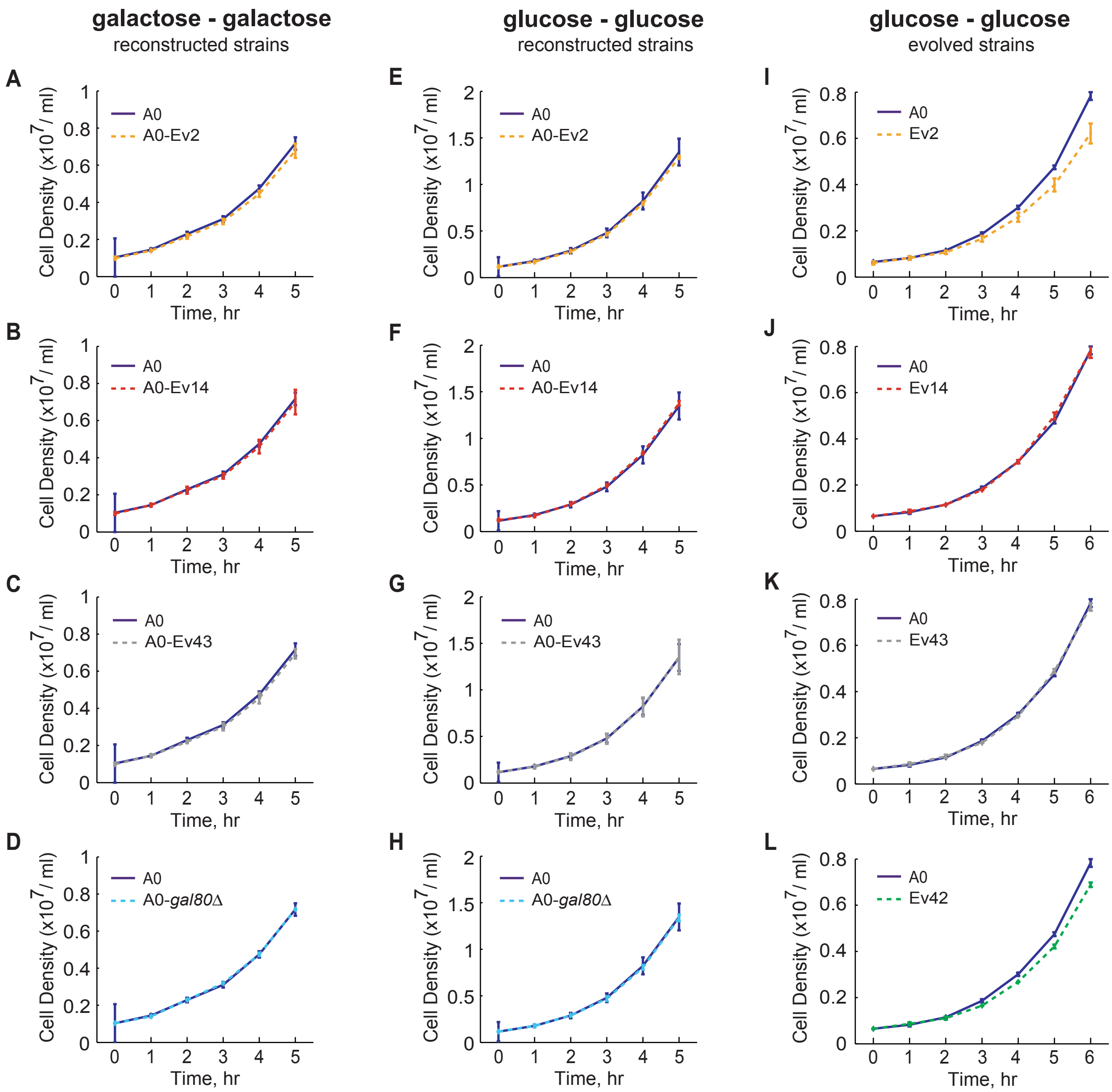
galactose - galactose
reconstructed strains
glucose - glucose
reconstructed strains
glucose - glucose
evolved strains
A
B
C
D
E
F
G
H
I
J
K
L
A0
A0-Ev2
A0-Ev14
A0-Ev43
A0-gal80Δ
Ev2
Ev14
Ev43
Ev42
Cell Density (x10^7 / ml)
Time, hr
