# Supplementary material for: High-Resolution Mutation Mapping Reveals Parallel Experimental Evolution in Yeast
Source: PLoS Biol. 2006 Jul 25;4(8):e256. doi: 10.1371/journal.pbio.0040256 (PMC1514788; doi:10.1371/journal.pbio.0040256)
Supplement: Supplementary file 8 [file pbio.0040256.sg008.pdf]

Figure S8

		36				158	
S.cerevisiae	... 31	WAIKTHYPAIL	41 ...	... 153	RKSPYILRAKE	163 ...
S.paradoxus	... 31	WAIKTHYPAIL	41 ...	... 153	RKSPYILRAKE	163 ...
S.mikatae	... 31	WAIKTHYPAIL	41 ...	... 153	RKSPYILRAKE	163 ...
S.bayanus	... 31	WAIKTHYPAIL	41 ...	... 153	RAKELISEGYI	163 ...
S.castelli	... 31	WAVKTHYPAIL	41 ...	... 153	RKSPYILRAKE	163 ...
K.lactis	... 31	WVAKTHFLAIQ	41 ...	... 154	RKSPYIVRAKE	163 ...
Mutation		Y			N	

		222			361	
S.cerevisiae	... 217	ILQYMTSSYFS	227 ...	... 356	RNYNAIVGNIH	366 ...
S.paradoxus	... 217	ILQYMTSSYFS	227 ...	... 356	RNYNAVVGNIH	366 ...
S.mikatae	... 217	ILQYMTCSYFS	227 ...	... 356	RNYNAVVGNIH	366 ...
S.bayanus	... 217	LLQYMTSSYFS	227 ...	... 356	RNYNAVVGNIH	366 ...
S.castelli	... 217	LLQYMTGSYFS	227 ...	... 351	RNYNAVNGNIF	361 ...
K.lactis	... 218	VLQYITGSYFQ	228 ...	... 372	RNYNSVVGNIL	382 ...
Mutation		K			M	

		392	
S.cerevisiae	... 387	SQFVMQGFDFE	397 ...
S.paradoxus	... 387	SQFVMQGFDFE	397 ...
S.mikatae	... 387	SQFVMQGFDFE	397 ...
S.bayanus	... 387	SQFVMQGFDFE	397 ...
S.castelli	... 382	SQFIMQGFEFE	392 ...
K.lactis	... 412	TKFDKQGFRFE	422 ...
Mutation		H	
