# Supplementary material for: High-Resolution Mutation Mapping Reveals Parallel Experimental Evolution in Yeast
Source: PLoS Biol. 2006 Jul 25;4(8):e256. doi: 10.1371/journal.pbio.0040256 (PMC1514788; doi:10.1371/journal.pbio.0040256)
Supplement: Supplementary file 9 [file pbio.0040256.sg009.pdf]

Figure S9

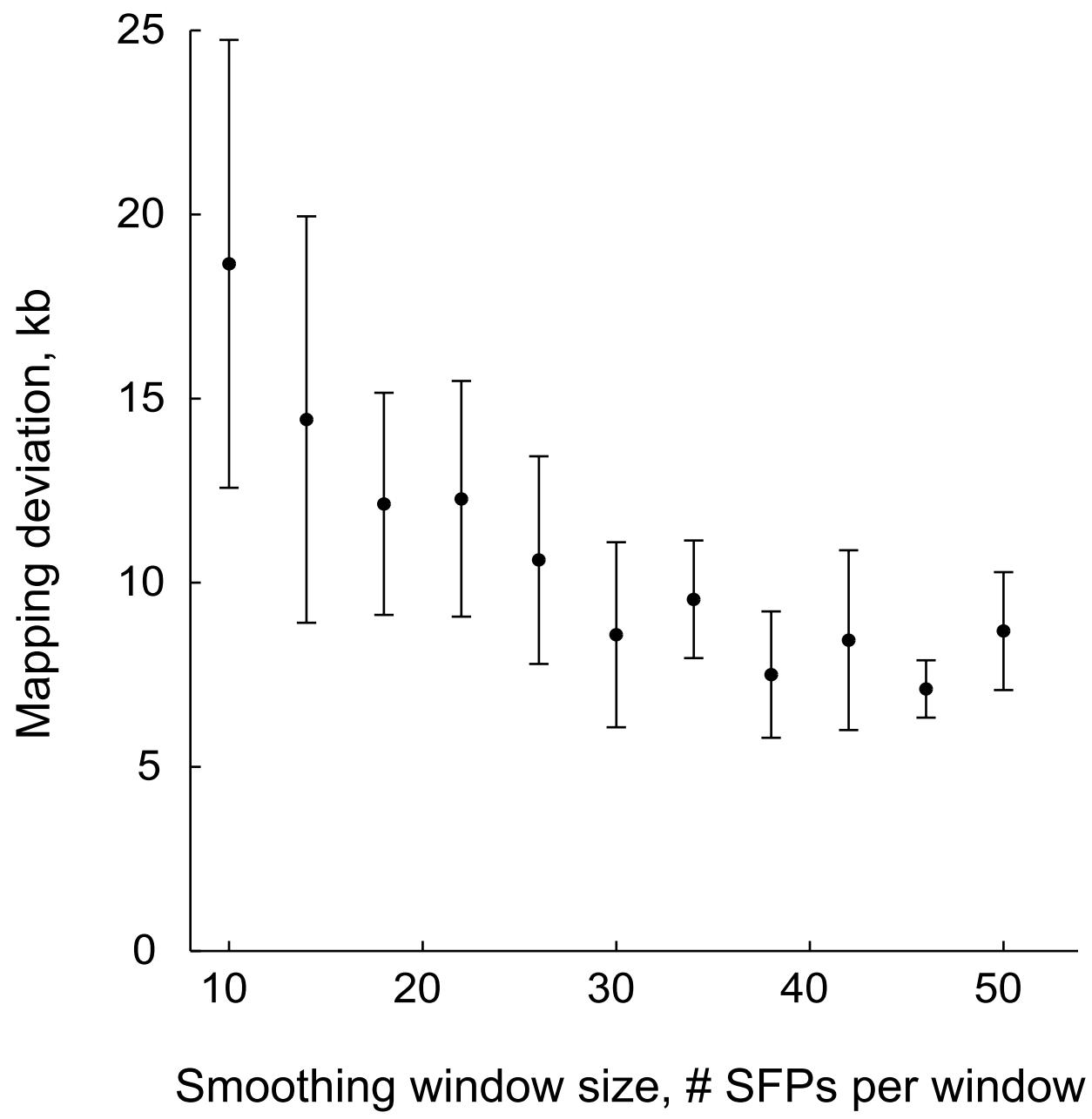
25
20
15
10
5
0
Mapping deviation, kb
10
20
30
40
50
Smoothing window size, # SFPs per window
