# Supplementary material for: High-Resolution Mutation Mapping Reveals Parallel Experimental Evolution in Yeast
Source: PLoS Biol. 2006 Jul 25;4(8):e256. doi: 10.1371/journal.pbio.0040256 (PMC1514788; doi:10.1371/journal.pbio.0040256)
Supplement: Supplementary file 10 [file pbio.0040256.sd001.doc]

**Text S1.** Factors that affect Linkage Map Score

The Linkage Map Score (LMS) is dependent on a variety of factors that may vary along the genome, including the level of enrichment of the selected locus in the selected pool of segregants, the local marker (SFP) density, the relative difference between the hybridization intensity of the target strain and that of the control pool, the coefficient of variation of the intensities between replicate arrays, the smoothing window size/type (which is kept constant across the whole genome unless stated otherwise), and local recombination rate. Thus, the LMS cannot be an indicator of the level of enrichment of a given allele in the selected pool or of its relative contribution to the trait. The Linkage Map Score can vary largely between peaks across the genome, as seen with our test case genes, *can1* and *LYS5*. However, the differences in LMS values between different peaks should not affect the mapping precisions of multiple quantitative trait loci (QTLs), since our algorithm for estimating the peak center is not affected by the peak’s height but rather by its shape. The LMS of one QTL could affect the score of another QTL only if they lie sufficiently close, a limitation of many QTL mapping methods. The relative value of LMS compared with the estimated significant peak cutoff is more important than the relative LMS values between peaks. Peaks may not pass the estimated significant peak cutoff for several reasons, including: (i) the linked QTL is less than 100% enriched in the selected pool, (ii) the QTL lies in a low SFP density region, (iii) the QTL lies in a region that is affected by one the factors mentioned above, or (iv) the peak is a false positive peak.
