# Supplementary material for: High-Resolution Mutation Mapping Reveals Parallel Experimental Evolution in Yeast
Source: PLoS Biol. 2006 Jul 25;4(8):e256. doi: 10.1371/journal.pbio.0040256 (PMC1514788; doi:10.1371/journal.pbio.0040256)
Supplement: Supplementary file 11 [file pbio.0040256.st001.doc]

**Table S1.** Mapping precision as a function of different array preprocessing methods

| **Gene** | **PM-MM** | **PM** | **log_10_PM** | **PM-b1** | **PM-b2** | **PM-b3** |
| --- | --- | --- | --- | --- | --- | --- |
| *KAN^R^* | 7.5 ± 6.4 | 8.2 ± 7 | 8.9 ± 7.6 | 8.2 ± 6.1 | 15.9 ± 5.9 | 7.9 ± 6.7 |
| *HYG^R^* | 4.3 ± 3.6 | 14.3 ± 10.5 | 9.6 ± 5 | 7.6 ± 5.5 | 9.4 ± 11.4 | 14.9 ± 10.5 |
| *NAT^R^* | 14.9 ± 8.6 | 12.9 ± 6.7 | 12.5 ± 4 | 10.2 ± 2.7 | 28.9 ± 6 | 12.2 ± 7 |
| *can1* | 1.7 | 2.1 | 1.1 | 3.1 | 15.1 | 2.1 |
| *LYS5* | 9.5 | 11.5 | 11.5 | 11.5 | 42.5 | 11.5 |
| **Mean mapping deviation** | **7.6** | **9.8** | **8.7** | **8.1** | **22.4** | **9.7** |

We tested the effect of different array preprocessing methods on mapping precision and found that our method is fairly insensitive to the method used. The mapping deviations of the predicted positions of the five test case genes from their real centers are given in kilobases. The 10,330 W303-SK1 SFPs identified using the PM-MM preprocessing method at a p-value cutoff of 10^-6^ were used for the comparison. The mean mapping deviations and standard deviations for *KAN^R^*, *HYG^R^*, and *NAT^R^* were calculated from three separate mapping experiments. To calculate a local background (b1, b2, b3) we divided the array into 10 by 10 squares and subtracted the following values from each PM: (i) median of the MM values lying in the square encompassing the given PM (b1); (ii) mean of the lower 5 percent of ranked PM and MM values in the corresponding square (b2); (iii) mean of the lower 2 percent of ranked PM values in the corresponding square (b3). These methods are similar to those used by the Affymetrix GeneChip software (<http://www.affymetrix.com/support/technical/whitepapers.affx>, Statistical Algorithms Description Document [57]) and Li and Wong’s dChip software [61]. All intensities were normalized by a median of a spatially local set of invariant PM values. For log_10_PM the logarithm of PM was divided by the median log_10_PM of the local invariant probes, similar to the approach used in previous single segregant mapping methods in yeast [22,24,25]. In the software of our mapping method, the user will have the option of choosing between different array preprocessing methods, including those that do not use the MM, and will be able to adjust tunable parameters, such as the percentile of ranked intensities used for background subtraction (http://www.cgr.harvard.edu/MutationMapping). This will allow users to find the optimal method for their mapping system.
