# Supplementary material for: High-Resolution Mutation Mapping Reveals Parallel Experimental Evolution in Yeast
Source: PLoS Biol. 2006 Jul 25;4(8):e256. doi: 10.1371/journal.pbio.0040256 (PMC1514788; doi:10.1371/journal.pbio.0040256)
Supplement: Supplementary file 12 [file pbio.0040256.st002.doc]

**Table S2.** The effect of using different array preprocessing methods for identifying Single-Feature Polymorphisms (SFPs) on mapping precision

|  | **PM-MM** | **PM** | **log_10_PM** | **PM-b1** | **PM-b3** |
| --- | --- | --- | --- | --- | --- |
| **# SFPs: (p<10^-6^)** | 10,330 | 10,958 | 11,517 | 9,327 | 10,782 |
| *KAN^R^* | 7.5 ± 6.4 | 8.2 ± 6.7 | 10.5 ± 6.1 | 9.3 ± 6.1 | 7.9 ± 6.8 |
| *HYG^R^* | 4.3 ± 3.6 | 1.9 ± 0.6 | 7.1 ± 1 | 5 ± 1.5 | 1.5 ± 0.6 |
| *NAT^R^* | 14.9 ± 8.6 | 12 ± 6.1 | 12.1 ± 5.5 | 17.5 ± 8.1 | 12 ± 5.1 |
| *can1* | 1.7 | 2.1 | 4.8 | 6.1 | 4.1 |
| LYS5 | 9.5 | 9.8 | 7.8 | 12.9 | 12.5 |
| **Mean mapping deviation** | **7.6** | **6.8** | **8.5** | **10.2** | **7.6** |

The mapping precision of our method is robust to the array preprocessing method used for calling features polymorphic (SFPs) at a p-value cutoff of 10^-6^. Different probe preprocessing methods were used to identify SFPs, and the linkage analysis was then done using the PM-MM preprocessing method. This allowed us to isolate the effect of the preprocessing method used to find SFPs on mapping precision. The mapping deviations of the predicted positions of the five test case genes from their real centers are given in kilobases. Although the true positive to false discovery rate ratios may vary between the different methods (data not shown), this does not appear to have a significant effect on the final outcome of the mapping. This is in accordance with the prediction of our simulations, that false SFPs do not have a significant effect on mapping precision (Figure 3C). The local backgrounds (b1, b3) are defined in Table S1. The mean mapping deviations and standard deviations for *KAN^R^*, *HYG^R^*, and *NAT^R^* were calculated from three separate mapping experiments.
