# Supplementary material for: High-Resolution Mutation Mapping Reveals Parallel Experimental Evolution in Yeast
Source: PLoS Biol. 2006 Jul 25;4(8):e256. doi: 10.1371/journal.pbio.0040256 (PMC1514788; doi:10.1371/journal.pbio.0040256)
Supplement: Supplementary file 13 [file pbio.0040256.st003.doc]

**Table S3. Mapping precision of test case genes as a function of array replicate number**

|  | Number of array replicates | | |  |
| --- | --- | --- | --- | --- |
| Test case gene | **2** | **3** | **4** | ***R^2^*** |
| *can1* | 6.9 | 3.3 | 1.7 | -0.98 |
| LYS5 | 5.7 | 6.7 | 9.5 | 0.97 |
| *KAN^R^* | 6.0 | 2.3 | 0.2 | -0.99 |
| *HYG^R^* | 14.9 | 13.5 | 8.3 | -0.95 |
| *NAT^R^* | 31.2 | 29.7 | 24.2 | -0.95 |
| Mean deviation | 12.9 | 11.1 | 8.8 | -1.00 |

The mapping deviations of the predicted gene locations from their actual centers are presented here as an average of the absolute deviations calculated from all possible combinations of duplicate, triplicate, and four replicate arrays out of four replicates from a single mapping experiment. Mapping deviation is given in kilobases. The number of array replicates refer to the selected pool, control pool, and the target strain, *W303*. *R^2^* denotes the correlation of coefficient of mapping deviation as a function of replicate number.
