# Supplementary material for: High-Resolution Mutation Mapping Reveals Parallel Experimental Evolution in Yeast
Source: PLoS Biol. 2006 Jul 25;4(8):e256. doi: 10.1371/journal.pbio.0040256 (PMC1514788; doi:10.1371/journal.pbio.0040256)
Supplement: Supplementary file 14 [file pbio.0040256.st004.doc]

##### Table S4. Genotype of yeast strains

| Strains isogenic with W303 | |
| --- | --- |
| JYL13 | *MATa ura3-1 his3-11,15 trp1-1 ade2-1 can1-100* |
| AVS4 | *MATa ura3-1 his3-11,15 trp1-1 ade2-1 can1-100 KAN^R^ HYG^R^ NAT^R^* |
| JYL243 | *bud5::FUR1::MATa::tsm1::LYS5* *his3-11,15 leu2-3,112 can1-100 trp1-1::TSM1::TRP1 ura3∆ lys5∆ fur1∆ trp5∆ msh2∆::KAN* |
| JYL246 | *bud5::URA3::MATα::tsm1::TRP5* *his3-11,15 leu2-3,112 can1-100 trp1-1::TSM1::TRP1 ura3∆ lys5∆ fur1∆ trp5∆ msh2∆::KAN* |
| Strains isogenic with SK1 | |
| JYL394 | *MATα ura3 (Δ SmaI-PstI) trp1::hisG leu2::hisG lys5Δ::3xHA ho::hisG GAL3* |
| JYL631 | *bud5::FUR1::MATa::tsm1::LYS5* *his3::hisG leu2::hisG trp1∆::TSM1::TRP1 ura3∆ lys5∆ fur1∆ trp5∆ flo8∆* |
| JYL632 | *bud5::URA3::MATα::tsm1::TRP5* *his3::hisG leu2::hisG trp1∆::TSM1::TRP1 ura3∆ lys5∆ fur1∆ trp5∆ flo8∆* |
| Other strains | |
| BY4742 | *MATα his3∆ leu2∆ lys2∆ ura3∆* |
| YJM789 | *MATα ho::hisG*  *gal2 lys2* |
